# Synthetic and Naturally Occurring Heterocyclic Anticancer Compounds with Multiple Biological Targets

**DOI:** 10.3390/molecules26237134

**Published:** 2021-11-25

**Authors:** Richard Kwamla Amewu, Patrick Opare Sakyi, Dorcas Osei-Safo, Ivan Addae-Mensah

**Affiliations:** 1 Department of Chemistry, School of Physical and Mathematical Sciences, College of Basic and Applied Sciences, University of Ghana, Legon, Accra P.O. Box LG 56, Ghana; ramewu@ug.edu.gh (R.K.A.); opsakyi@st.ug.edu.gh (P.O.S.); dosei-safo@ug.edu.gh (D.O.-S.); 2 Department of Chemical Sciences, School of Sciences, University of Energy and Natural Resources, Sunyani P.O. Box 214, Ghana

**Keywords:** homeostasis, metastasis, angiogenesis, apoptosis, vascularization, heterocyclic compounds, multiple biological targets

## Abstract

Cancer is a complex group of diseases initiated by abnormal cell division with the potential of spreading to other parts of the body. The advancement in the discoveries of omics and bio- and cheminformatics has led to the identification of drugs inhibiting putative targets including vascular endothelial growth factor (VEGF) family receptors, fibroblast growth factors (FGF), platelet derived growth factors (PDGF), epidermal growth factor (EGF), thymidine phosphorylase (TP), and neuropeptide Y4 (NY4), amongst others. Drug resistance, systemic toxicity, and drug ineffectiveness for various cancer chemo-treatments are widespread. Due to this, efficient therapeutic agents targeting two or more of the putative targets in different cancer cells are proposed as cutting edge treatments. Heterocyclic compounds, both synthetic and natural products, have, however, contributed immensely to chemotherapeutics for treatments of various diseases, but little is known about such compounds and their multimodal anticancer properties. A compendium of heterocyclic synthetic and natural product multitarget anticancer compounds, their IC_50_, and biological targets of inhibition are therefore presented in this review.

## 1. Introduction

Cancer is a complex group of diseases the onset of which is characterized by abnormal cell division with the potential of spreading to other parts of the body over time [1,2]. The abnormal cell differentiation is normally accompanied by drastic weight loss, prolonged cough, and abnormal lump growth [3]. The prevalence of cancer is spiraling globally. Currently, cancer ranks second as the leading cause of death worldwide and in the year 2020 alone, an estimated 19.3 million new cases with 10 million deaths were recorded [4]. These statistics translate into an annual death rate that is a little over 50% of the annual new cases. Cancers of the stomach, lung, breast, prostate/cervix, and colorectum have all been reported but the most frequently diagnosed are lung and breast cancers followed by prostate which occurs predominantly among the aging male population [5]. Sex and age have become of paramount importance in cancer susceptibility and treatment, and men are said to be more prone to infections than women [6,7].

Prior to cancer becoming metastatic, its spread is initiated by pro-angiogenic factors consisting of vascular endothelial growth factor (VEGF) receptors, fibroblast growth factor (FGF), platelet derived growth factors (PDGF), epidermal growth factor (EGF), thymidine phosphorylase (TP), neuropeptide Y4 (NY4), and platelet factor 4 (PF4), etc. [8,9]. Upon initiation, the survival of these cancer cells and their proliferation are dependent on the supply of oxygen, nutrients, and clearance of waste products [10,11]. All these processes are dependent on the sprouting of vascular networks. New blood vessels form through a process called angiogenesis [12]. In the absence of the vascular extension, cancer cells become necrotic or undergo a process called apoptosis, limiting the proliferation rate [10]. The level of expression of necrotic or apoptotic factors is a reflection of the aggressiveness of a tumor [13,14]. Generally vascular development starts by basement membrane destruction which leads to the release of angiogenic factors. This activates endothelial cells to migrate which then proliferate and stabilize until a safeguard sets in [10,15,16]. Regulation of neoplastic vascularization as an important step in limiting cancer proliferation has received immense attention and extensive research [8,12]. To starve these tumor cells, cautious targeting of vessel sprouting activators with a foreknowledge of how they control this chemical signal is crucial in maintaining homeostasis [17,18].

Numerous cancer targets including VEGF, FGF, PGF, EGF, PDGF, topoisomerase I and II, histone deacetylase, tyrosine kinase, and transforming growth factor-alpha (TGF-α) have been elucidated with multiple studies focused mainly on targeting the initiator VEGF [1,19,20,21]. However, halting VEGF signaling has not been very effective as reports of disease progression after treatment have become rampant [22].

**Figure 1:**
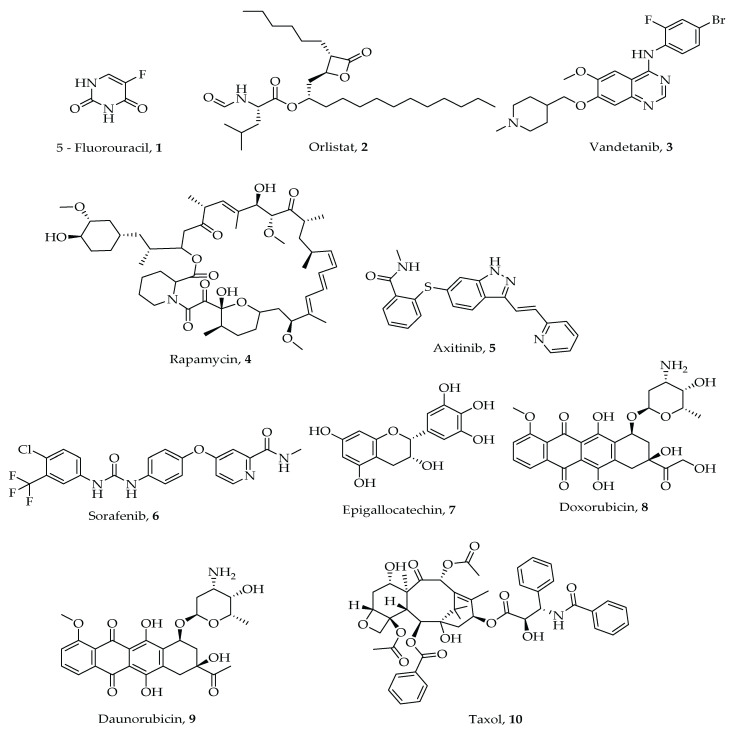
Chemical structures of some heterocyclic compounds used for treating various types of cancer.

A number of heterocyclic anticancer agents both synthetic and naturally occurring are in use and more are still being sought after [23,24,25,26]. Some examples are as shown in Figure 1. Heterocyclic compounds (ring compounds containing C and any of the atoms N, O, and S) have been explored for their medicinal properties for the treatment of various diseases including cancer. Introduction of these heteroatoms improves solubility, polarity, and hydrogen bonding abilities leading to ADMET (Adsorption, Distribution, Metabolism, Excretion, and Toxicity) optimization of druggable candidates. Therapeutic heterocyclic agents such as 5- fluorouracil **1**, orlistat **2**, vandetanib **3**, rapamycin **4**, axitinib **5**, sorafenib **6**, epigallocatechin **7**, doxorubicin **8**, daunorubicin **9**, and Taxol **10** (Figure 1) have demonstrated high inhibition against different types of cancer cells [27,28,29].

**Scheme 1:**
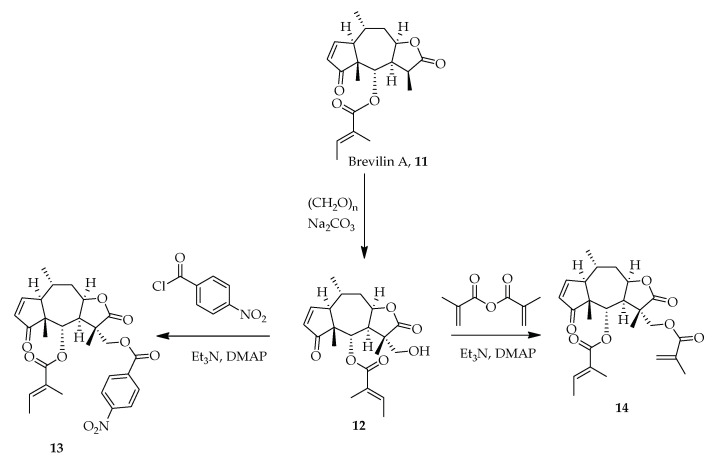
Synthetic routes of Brevilin A analogues [26].

Brevilin A, **11**, a heterocyclic sesquiterpene lactone natural product isolated from *Centipeda minima* exhibits anticancer properties [30]. Studies have shown that Brevilin A attenuates the signal transducer and activator of transcription (STATS 3) and Janus kinase and tyrosine kinase activity thereby inhibiting cell growth, inducing apoptosis and reducing cell metastasis [31]. Lee, Chan et al., synthesized analogues of Brevilin A and found that **13** and **14** exerted greater anticancer properties than **11**. Aldol reaction of **11** and paraformaldehyde in the presence of sodium carbonate produced **12**. Acetylation of **12** with *p*-nitrobenzoyl chloride and methacrylic anhydride afforded **13** and **14**, respectively (Scheme 1) [26].

Challenges associated with cancer treatments such as drug resistance, systemic toxicity of administered drugs, and drug ineffectiveness are widespread [32,33]. Furthermore, confounding factors such as the multiple signaling nature of pathways and the propensity of most cancer cells to mutate have hindered the search for an effective therapeutic agent for combating cancers, calling for urgent search to identify new anticancer agents as drug leads [19,34]. To overcome these challenges, multi-target heterocyclic inhibitors are proposed as an option in achieving success in the fight against various forms of cancers.

**Figure 2:**
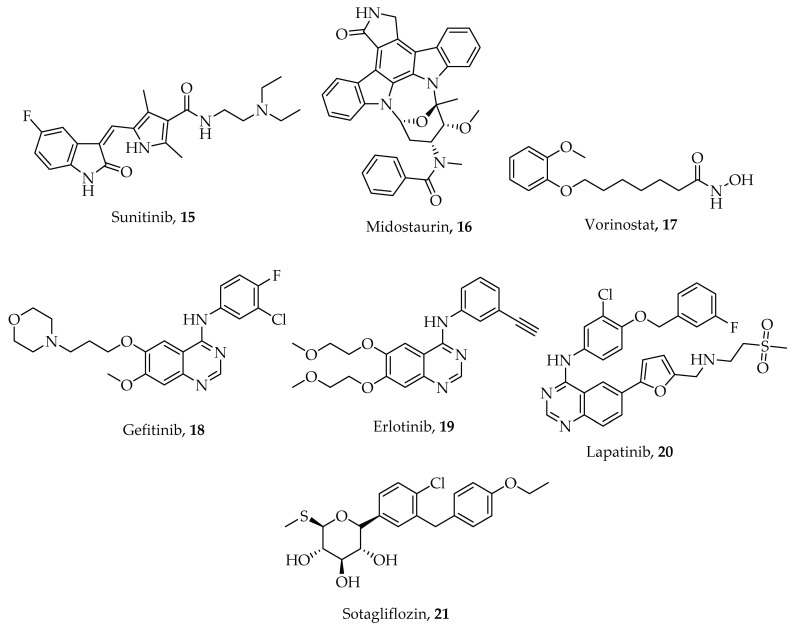
Chemical structures of some heterocyclic multi-target anticancer agents. Compounds **15–17** are in clinical use while **18–21** are in various stages of clinical trials.

Emerging heterocyclic compounds with demonstrable anticancer activities include sunitinib **15**, midostaurin **16**, and vorinostat **17** (Figure 2). They possess multi-regulatory activity against growth factors like vascular endothelium growth factor receptor (VEGFR), platelet-derived growth factor receptor alpha (PDGFRA’s c-Kit) and tyrosine kinase 3 (FLT-3) [35]. In the same vein, gefitinib **18**, erlotinib **19**, lapatinib **20**, and sotagliflozin **21** (Figure 2) with magnificent inhibitory potentials against human epidermal growth factor receptor 1 (HER1) and 2 (HER2) are also in phase 3 clinical trials confirming that multi-targeted therapy has a future [36,37].

This review therefore seeks to highlight the various types of heterocyclic multimodality anticancer agents (synthetic and natural products) and their biological targets with some emphasis on their mechanism of action. Various systematic rigorous methodological approaches were used to search literature to identify, collate, and critically appraise a body of previously published works relevant to the topic. The search comprised of putting relevant key words into Scifinder, Scopus, Google scholar, PubMed, and others and appraising them for their suitability. Original hard copy offprints of published papers in our various personal archives were also consulted for relevant information [38,39].

## 2. Heterocyclic Compounds

Heterocyclic organic compounds are designated as integral components on a wide array of structures with both pharmacological and biological importance. They constitute a large cohort of structures with immense importance in the life sciences. Their diverse characteristics include the display of a wide variety of intermolecular interactions, different ring sizes, their planarity if aromatic, and functional group versatility. Among the twenty amino acids, proline, histidine, and tryptophan contain heterocyclic rings. Furthermore, the basic units that contain instructions for development, growth, and reproduction are all made of a heterocyclic nucleus.

**Figure 3:**
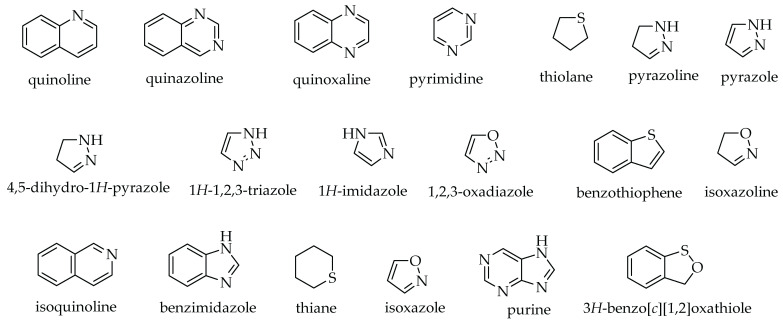
Examples of heterocyclic chemotypes.

Many natural products such as alkaloids, flavonoids, terpenoids, coumarins, anthocyanins, isothiazoles, etc., contain heterocyclic rings, possess a broad spectrum of activities against numerous disease-causing organisms. In addition, recent structural assessment of heterocyclic moieties among synthetic compounds by Marson has revealed that their incorporation plays a very significant role in drug-likeness and target binding by limiting toxic metabolite production, increasing water solubility, and lowering conformational entropy [40]. Heterocyclic chemotypes like quinoline, quinazoline, quinoxaline, pyrimidine, pyrazoline, 1,2,4-triazole, imidazole, benzimidazole, isoxazoline, isoquinoline, pyrazole, and isoxazole (Figure 3) can be found in compounds used in the treatment of debilitating diseases like malaria, tuberculosis, cancer, neuro-degenerative diseases, fungal, and bacterial infections [41,42,43]. The following are heterocyclic compounds that, if present as moieties in synthetic or naturally occurring compounds, may confer anti-cancer properties. 

### 2.1. N-Based Multi-Target Anticancer Heterocycles

*N*-based heterocyclic compounds have attracted much interest and attention from medicinal chemists and biologists. Their broad range of biological activities and wide applications in the design of drugs with exceptional selectivity for DNA via hydrogen bonding interaction has contributed to their high propensity for conferring anti-cancer properties on various synthetic and natural compounds in which they occur [44].

#### 2.1.1. Synthetic, Semi-Synthetic and Hybrid N-Heterocycles

**Figure 4:**
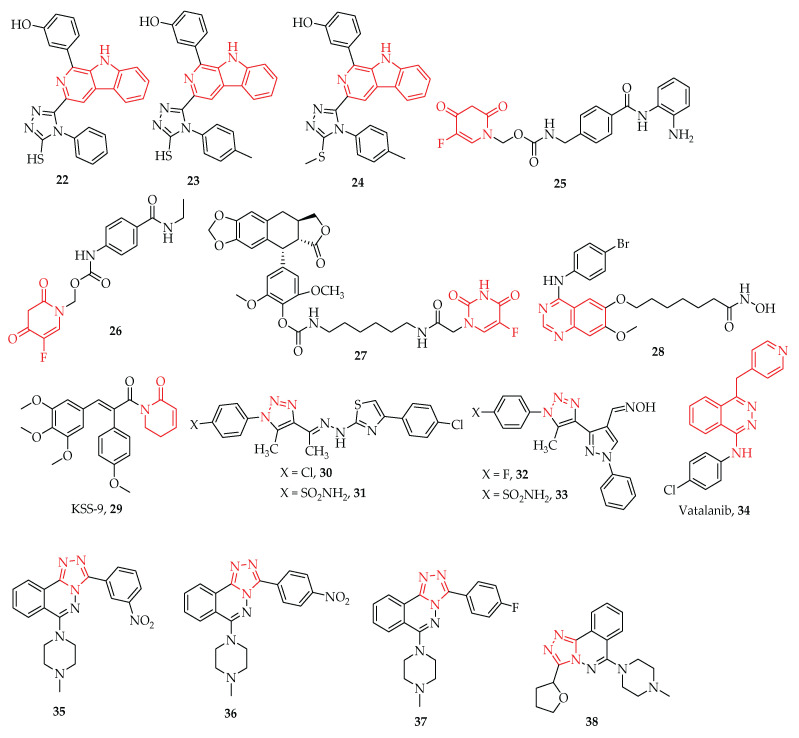
Examples of synthesized multi-target anticancer *N*-based heterocyclic compounds.

*N*-based heterocycles such as **22**, **23**, and **24** (Figure 4) possess multi-target cancer inhibitory activity. They cause antagonism at 9.4 μM (compound **22**), 2.50 μM (compound **23)**, and 9.30 μM (compound **24**) against topoisomerase 1. The compounds also antagonize kinase spindle protein (KSP) at 28.58, 8.91, and 19.47 μM, respectively [45]. Pathway analysis has suggested that the anticancer properties of the compounds are due to G1 apoptosis and cell cycle arrest at the G2/M phase. It has further been observed that the selectivity and strong binding of the three compounds are partly due to the planarity of carboline and phenolic OH groups [45].

Additionally, cell studies of the combined histone deacylase (HDAC) inhibitors **25** and **26** with fluorouracil, an isosteric replacement for pyridine against HDAC protein source (Hela cell nuclear extract), resulted in inhibitory potentials of 5.92 and 2.31 μM for compounds **25** and **26** compared to the moderate anti-proliferative activity estimated with seven tumor cell panels (K-562, A549, U266, PC-3, HCT-116, ES-2, and HL-7720) [46]. Discovery of the essential role of angiogenesis in tumor progression by Judah Folkman in the late 20th century has been recently supported by many genetic and epidemiological studies [47]. Similarly, a combination of deoxy-podophyllotoxin and 5-fluorouracil-yl showed an increased cytotoxic activity against tumor cell lines compared with the anticancer drugs VP-16 and 5-FU [48].

4-Deoxypodophyllotoxin-5-fluorouracil, **27**, induced cell-cycle arrest in the G2/M phase by regulating levels of cdc2, cyclinB1, and p-cdc2 in A549 cells and migration of A549 cells via down-regulation of matrix metallopeptidase 9 (MMP-9) and up-regulation of tissue inhibitor of metalloproteinase (TIMP-1) [48]. A multi-target inhibitor is also formed by fusing vandetanib **3**, a vascular endothelial growth factor receptor 2 (VEGFR2) inhibitor with Vorinostat, **17**, an HDAC inhibitor. The fused product, **28** exhibited a very potent inhibitory activity against HDAC with an IC_50_ of 2.2 nM and strong inhibitory effect against VEGFR-2 at 74 nM [49]. Its inhibitory activity against a human breast cancer cell line MCF-7 was at IC_50_ of 0.85 μM [49]. Arylation of piperlongumine, an amide alkaloid present in *Piper longum*, *Piper guineense* and other *Piper* species, with combrestatin A4 afforded a potent semi-synthetic and hybrid compound KSS-9, **29**, which exhibited increased reactive oxygen substrate (ROS) generation, tubulin depolymerization, and G2/M cell cycle phase block [50].

The association between inflammation and tumor progression exemplified by inflammatory bowel diseases, bronchitis and prostatitis has reshaped the focus of many anticancer drug design programs from single targeting towards the use of multi-targeting-directed ligands. Of the seventeen nitrogen-containing heterocyclic compounds **22**–**38**, four of them (**30**, **31**, **32**, and **33**) which are 1,2,3-triazole derivatives, were potent against cyclooxygenase-2 (COX-2) and 15-lypoxygenases (15-LOX) at sub-micromolar and micro molar concentrations, respectively, with only **31** and **33** showing activity against tumor associated carbonic anhydrase enzymes [51].

Despite the few pyridazines found in nature, recent demonstration of potency coupled with low toxicity have generated interest within the scientific community. An example is the VEGFR inhibitor vatalanib, **34**, which is currently in clinical trials [52]. Investigating the effects of aryl group substitution on the 1,2,4-triazolo core, Romero and coworkers reported that four compounds **35**–**38** (Figure 4) exhibited good IC_50_ activities against breast cancer cells below 12 μM [53]. In contrast to the breast cancer cell lines, lung carcinoma cells A549 were resistant with only **35**, **36**, and **38** being moderately cytotoxic (13.98 to 20.40 μM). However, **36** and **37** exhibited moderate anticancer activities against breast MCF-7 and SKBr3 carcinomas with bioactivities of 5.55 (**36**) and 11.13 μM (**37**) for MCF-7 and 5.85 (**36**) and 8.48 μM (**38**) for SKBr3. While compound **35** showed moderate anticancer activity (8.58 μM), compound **36** exhibited strong anticancer activity against HeLa cell line at 0.54 μM [53]. Structural extrapolations identified 3-nitrosubstitution on the aryl ring to be the most convenient pharmacophore with strong affinity towards EGFR tyrosine kinase enzyme [53].

**Figure 5:**
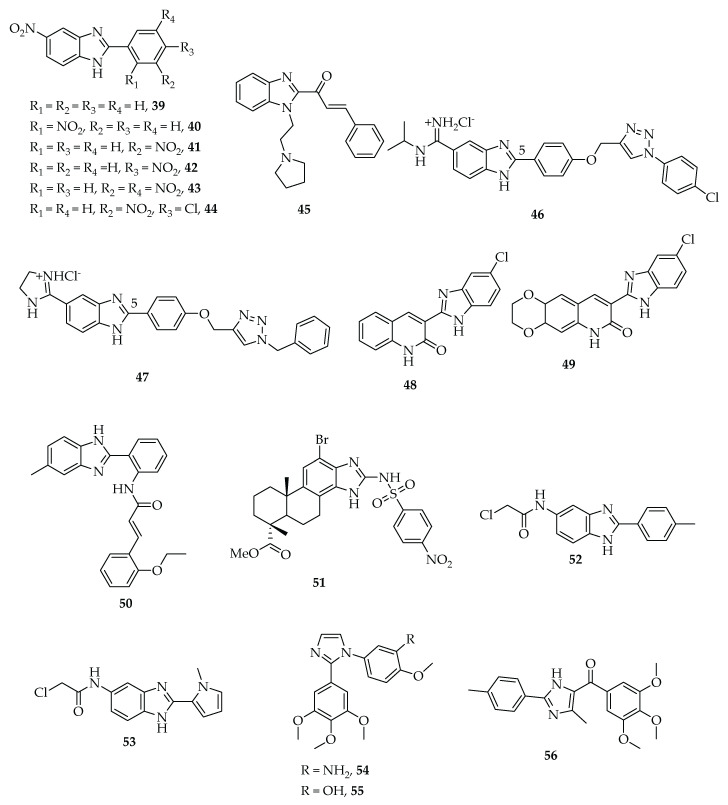
Examples of synthesized imidazole and benzimidazole multi-target anticancer compounds.

A number of biologically active molecules with imidazole and benzimidazole nuclei have been explored for their diverse therapeutic uses including, but not limited to anti-parasitic, antimicrobial, antiviral, anti-helminthic, anti-hypertensive, analgesic, anti-inflammatory, anti-ulcer, antiallergic applications [54]. Romero-Castro et al., 2011 synthesized six novel 2-aryl-5(6)-nitro-1H-benzimidazole derivatives (**39**–**44**) (Figure 5) and observed that the cytotoxicity of these compounds against various cancer cells was enhanced by the introduction of a nitro group at position 2 or 3 of the phenyl ring [55]. Among the derivatives, compound **44** was the most cytotoxic agent against all the cancer cell lines displaying an IC_50_ less than 10 μM. Interestingly, the same compound **44** was less cytotoxic against the non-neoplastic HACAT cell line. The mode of action exhibited by these compounds involves growth suppression via PARP inhibition, attenuation of DNA replication followed by S phase cell cycle arrest resulting in apoptosis [55]. Similarly, evaluation of the activity of the benzimidazole derivative **45** against Hep3B-SR and HuH7-SR cell lines revealed that at 25 μM, it exhibited potent antiproliferative effects on the two cell lines [56]. It perpetuates its mode of action by inhibiting AKT, STAT3, and PARP as well as downregulating Fas, at the cell surface [56].

In another study, novel amidino 2-substituted benzimidazole derivatives linked to 1,4-disubstituted 1,2,3-triazoles by phenoxy methyl or ethyl linkers were synthesized [57]. An in vitro antiproliferative screening of the series revealed that compounds **46** and **47** were the most potent against the cell lines displaying an IC_50_ in the micromolar range [57]. SAR study revealed that introduction of isopropyl and phenoxymethyl groups at C-5 of the benzimidazole ring enhanced the antitumor activity of the derivative. These novel compounds inhibited protein kinases including TGM2, CDK9, SK1, and p38 MAPK [57].

Benzimidazole–quinolinone is another class of *N*-based heterocycles that has attracted a lot of interest from medicinal and synthetic organic chemists possibly due to the presence of benzimidazole and quinolinone moieties [58]. An in vitro study evaluated twenty novel benzimidazole–quinolinone derivatives against HepG2, SKOV3, NCl-H460, and BEL-7404 cancer cell lines as well as HL-7702 normal liver cell line [59]. Two of the compounds, **48** and **49** displayed strong potency against HepG2 and BEL-7404 cell lines with IC_50_ values of 8.45 and 9.06 μM, respectively, by not only inhibiting CDK and PARP but also activating p53 protein, caspase-3 and 9 as well as upregulating of Bax [59].

Cinnamic acids are natural products known for their wide pharmacological activities [60]. Employing the pharmacophore-based design approach, a new hybrid **50** was synthesized from imidazole and cinnamic acid and its activity evaluated against lung and breast cancer cell lines [61]. In addition to inhibiting tubulin polymerization and arresting G2/M phase of the cell cycle, the compound displayed its cytotoxicity against all the cell lines with IC_50_ in the range of 0.25 to 1.5 μM [61]. Using a similar approach, Li et al. synthesized a series of imidazole derivatives of dehydroabietic acid and found them to exhibit antitumor activities via cell cycle arrest at the S phase, activation of intracellular ROS and decrease in mitochondrion potential leading to apoptosis [62]. Among the series, compound **51** exhibited the highest activity against MCF-7 (0.87 μM), HeLa (9.39 μM), and HepG2 cell lines (8.31 μM) [62].

Li et al., screened a cluster of benzimidazole compounds against receptor tyrosine kinase involved in vascular sprouting and found two novel inhibitors **52** and **53** with strong multi-targeting potentials against estimated glomerular filtration rate (EGFR), vascular endothelial growth factor (VEGFR) and platelet-derived growth factors (PDGFR) depicted by apoptosis and cell cycle arrest at G0/G1 stage [63]. Following their earlier success, they synthesized 1-substituted-2-arylimidazoles and tested them against seven cancer cell lines [64]. Of all the imidazoles synthesized, compound **54** exhibited the highest potency against all the cell lines with IC_50_ less than 200 nM [64]. Interestingly, compound **55** with an -OH group instead of -NH_2_ as in compound **54** also showed good potency against HeLa and HCT-15 cell lines with IC_50_ of 100 and 200 nM, respectively. When SAR was performed on the compounds, it was observed that while the addition of an aromatic ring on the imidazole ring improved the antiproliferative effects, the placement of an aliphatic group on the imidazole nitrogen and the replacement of the imidazole ring with an ester or amide caused a loss in activity [64]. The novel imidazole derivatives inhibit microtubules disrupting the formation of mitotic spindles which in turn causes cell cycle arrest and apoptotic cell death [64].

In an attempt to improve efficacy and overcome multidrug resistance, Bai et al. reported the imidazole derivatives **54**, **55** and **56**. Compound **56** was similar to **54**, but had an electron withdrawing group (carbonyl group) attached to the imidazole ring [65]. They reported that compound **56** targeted tubulin and DNA by disrupting microtubule assembly, causing DNA damage, and inducing cell cycle arrest. In addition, **56** mediated ROS mitochondrial and apoptotic pathways with IC_50_ of 27.42, 23.12, and 33.14 nM against SW480, HCT-116, and Caco2 cell lines, respectively [65].

**Figure 6:**
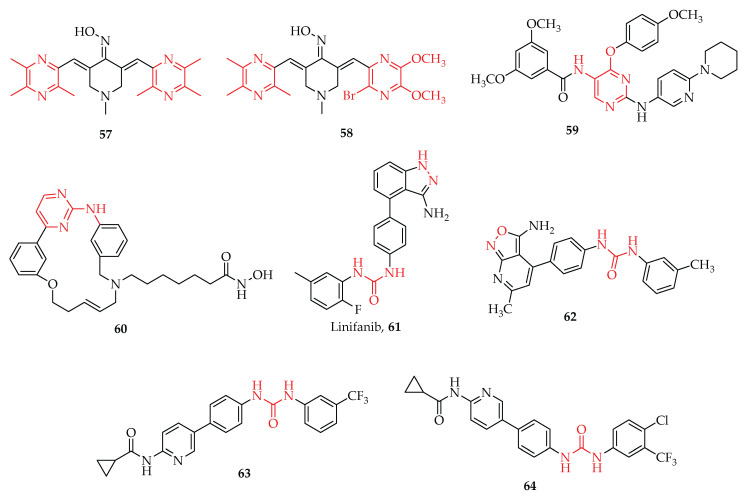
Derivatives of ligustrazine (**57**,**58**), 5-amino-4-pyrimidinol (**59**), *N*-(2-aminophenyl) benzamide acridine (**60**), Linifanib (**61**) and its isoxazol[3,4-b]pyridine-3-amino-derivative **62**, as well as other biphenyl-aryl urea derivative (**63** and **64**).

Building on a bioactive ingredient (ligustrazine) from widely used Chinese herbs, Zha et al. profiled the potentials of thirty-two derivatized ligustrazine frameworks against five cancer cell types, namely, tubulin, EGFR, tyrosine receptor kinase (TRK), kafirin (KAF), and B-*raf* proto-oncogene (BRAF) [66]. Among the synthetic candidates only two, **57** and **58** (Figure 6), exhibited potency as multi-target anticancer inhibitors to Tubulin, EGFR, KAF and BRAF and tropomyosin receptor kinase (TRK) as well as modulators of multidrug regulators (MDR) [66]. 

A demonstration of weak in vitro/in vivo activity by a hit compound is often exploited in the selection of an initial scaffold or starting material for the synthesis of improved bioactive compounds. Employing this approach, Farag et al. synthesized seven derivatives of 5-amino-4-pyrimidinol of which compound **59** showed broad spectrum anti-cancer activity, with the indicated percentage inhibitions of the following cancers: hematological (84.1%), colon (72,15%), CNS (66.34%), melanoma (66.48%), ovarian (51.55%), renal (55.95%), prostate (61.85%), and breast (60.87%) [67]. (*N*-(2-aminophenyl) benzamide acridine is another chemotype whose derivatives have been found to show improved suicidal action on tumor cells [68]. However, of the eleven novel synthetic *N*-heterocyclic macrocyclic derivatives tested (Figure 6) only compound **60** possessed multi-targeting potential against HDAC (87 nM), transmembrane ligand-activated receptor tyrosine kinase (FLT3) (87 nM), and Janus kinase 2 (JAK2) (0.68 μM), with a high cytotoxic effect on human acute myeloid leukemia cell line MV4-11 and human erythroleukemia (HEL) cells. Compound **60** acts by halting cell proliferation (IC_50_ 0.12–0.35 μM) triggered by G0/G1 cell cycle arrest and cellular apoptosis by inhibition of Topoisomerase 1 and HDAC [55].

(*N*-(2-aminophenyl) benzamide acridine is another chemotype whose derivatives have been found to show improved suicidal action on tumor cells [68].

Receptor tyrosine kinases (RTK) plays a critical role in the development and progress of cancers. Its inhibition has been found to stop transformation, proliferation, migration, differentiation, and metastasis of cancer cells. To improve the efficacy of Abbott’s Linifanib, **61** Shi et al. reported a diaryl-urea with isoxazol[3,4-b]pyridine-3-amino-derivative **62** with inhibition potentials of 4 nM, 3 nM, and 8 nM against fms related receptor tyrosine kinase 3 (FLT3), kinase insert domain receptor (KDR), and platelet-derived growth factor receptor beta (PDGFR-β), respectively [69,70]. In another study, Jinfeng Wang and co-workers discovered two triple inhibition chemotypes, **63** and **64** (Figure 6) from screening of these compounds derived from reaction between biphenyl-aryl urea and salicylaldoxime [71]. These two derivatives showed strong potency against pro-angiogenic receptors (VEGFR-2, tyrosine kinase 2 (TIE-2), and ephrin type B receptor (EphB4) resulting in the inhibition of endothelial cell survival, vascular permeability, migration, and proliferation [71].

#### 2.1.2. Natural Products from Various Sources

The medicinal use of natural products dates to ancient times [72,73]. Currently, it is estimated that about 70% of new drugs for the treatment of infectious diseases originate directly or indirectly from natural products of both floral and faunal origins [74,75]. Over the years there have been several reviews on plants as real and potential sources of compounds and their use in the treatment of cancer. Prominent among these reviews is the proceedings of the 16th annual meeting of the Society for Economic Botany: 1975 [76]. Cancer treatment has benefited immensely from natural products. Prominent among these are the *vinca* alkaloids from *Catharanthus roseus (Vinca rosea)* used for the treatment of leukemia and Taxol from *Taxus baccata* that is used for the treatment of cervical cancer [77,78]. The search for multitarget anticancer compounds can therefore not be complete without considering natural products from plant, soil, marine, fungal, and animal sources.

#### 2.1.3. Nitrogen-Heterocyclic Natural Products with Multitarget Inhibitory Properties

**Figure 7:**
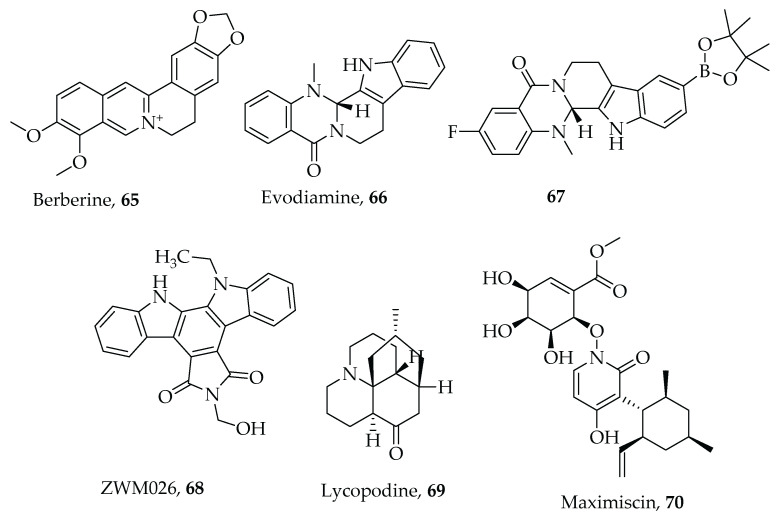
*N*-heterocyclic natural product compounds with multi-target effects on cancer cells.

The properties of some natural products have also been harnessed in the search for improved anticancer activities (Figure 7), For example, berberine **65**, isolated from *Rhizoma Coptidis* is a heterocyclic alkaloid known to have multi-targeting potentials against a myriad of human cancer receptors [79]. It suppresses growth of cholangiocarcinoma cell lines. This anticancer activity has been attributed to inhibitions of ERK1/2, NF-κB and STAT3 pathways. The compound also arrests the G1 cell cycle at 9.3 µM concentration within 48 h and 3.0 µM within 72 h [80]. Despite the DNA intercalation property of berberine **65** Lin and co-workers evaluated it on *N*-acetyltransferase (NAT) suppression in HL-60 cells and observed no effects on its gene expression at 30 µM [81].

Evodiamine, **66**, isolated from *Evodia rutaecarpa*, has been described as a tumor inhibitor with multi-targeting profiles against Topoisomerase I and II. Though the in vitro activity was encouraging, it demonstrated low in vivo potency [82]. Xinglin Li and co-workers have reported improved in vivo antitumor activity in a series of novel boron-incorporated derivatives of evodiamine **66** using HCT116, MCF-7, and A549 cell lines [83]. The N13 deprotected phenyl boronic acid derivative, **67** showed excellent antitumor activity in an HCT116 xenograft model in mice (IC_50_ of 16 nM) disrupting the PI3K/AKT signaling pathway [83].

Some naturally occurring mangrove indolocarbazoles were investigated for their multiple kinase inhibition potential against non-small-cell lung cancer cells. Compound **68** demonstrated selectivity towards oncoproteins HER2 (IC_50_ 190.7 nM), HER3 (IC_50_ 160.3 nM), and HER4, (IC_50_ 180.1 nM) [84]. It induced apoptosis and exerted a synergistic effect by combining with cisplatin in NCI- H1975 cells. It also potently and selectively inhibited growth of naturally occurring non-small-cell lung carcinoma (NSCLC) cells and constructed NIH-3T3 cells. Evaluation of the in vivo performance revealed that **68** inhibited EGFR-L858R/T790M with IC_50_ of 279.6 nM and RET (IC_50_ 183.7 nM) [84]. In addition to the potency of **68** against these targets, it also displayed downregulation of phosphor-Akt and phosphor-ERK [84].

Lycopodine, **69**, the main alkaloid of *Lycopodium clavatum* extract was reported to have anticancer effect on HeLa cells through DNA damage, 5-LOX inhibition, G0/G1 cell cycle arrest, deactivation of oxidation resistance (OXR) receptor and mitochondrion membrane depolarization [85]. J. Robles and co-workers identified maximiscin **70** as possessing multitarget anticancer properties [86]. They investigated its activity against cancer cell lines in vitro and in a xenograft mouse model of melanoma and found that **70** inhibited proliferation of all of the following five triple-negative breast cancer cell lines at the stated IC_50_ values: MDA-MB-468 (0.6 µM), MDA-MB-231 (39 µM), MDA-MB-453 (15 µM), HCC-70 (60 µM), and BT-549 (15 µM) [86]. It is suggested that the mechanism of action of the compound involves the induction of DNA damage as the primary suicidal action as well as activation of DNA damage response pathways and cell cycle arrest [86].

**Figure 8:**
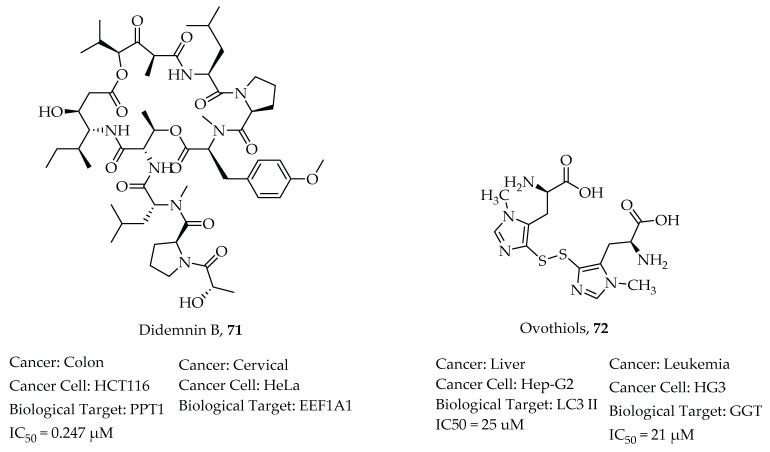
Chemical structures of Didemnin B, **71** and Ovothiols, **72** with a summary of their biological activity towards selected targets.

Didemnins are depsipeptides isolated from the sea squirt. They possess activity against various cancers including P388 lymphocytic leukemia and B16 melanoma [87]. Didemnin B, **71** is reported to target DNA functionality [88]. It perturbs cell-cycle and inhibits the synthesis of DNA at the elongation phase via the dual inhibition of palmitoyl-protein thioesterase 1 (PPT1) and eukaryotic translation elongation factor-1α (EEF1A1) [89]. It also activates caspases, thereby inducing apoptosis and inhibits protein synthesis by preventing eukaryotic elongation factor (Eef-2)-dependent translocation (Figure 8) [90].

Compound **72** is an example of a group of histidine-derived compounds called Ovothiols. These compounds are found in most members of the kingdom *Protoctista* (especially algae and protozoa) as well as many marine invertebrates including *Paracentrotus lividus*, *Strongylocentrotus purpuratus*, *Arbacia lixula*, *Marthasterias glacialis*, *Astropecten aurantiacus*, *Octopus vulgaris*, *Loligo vulgaris* and *Platynereis dumerilii* [91]. Compound **72** exhibits its anticancer properties by inhibiting autophagy initiation [92].

**Figure 9:**
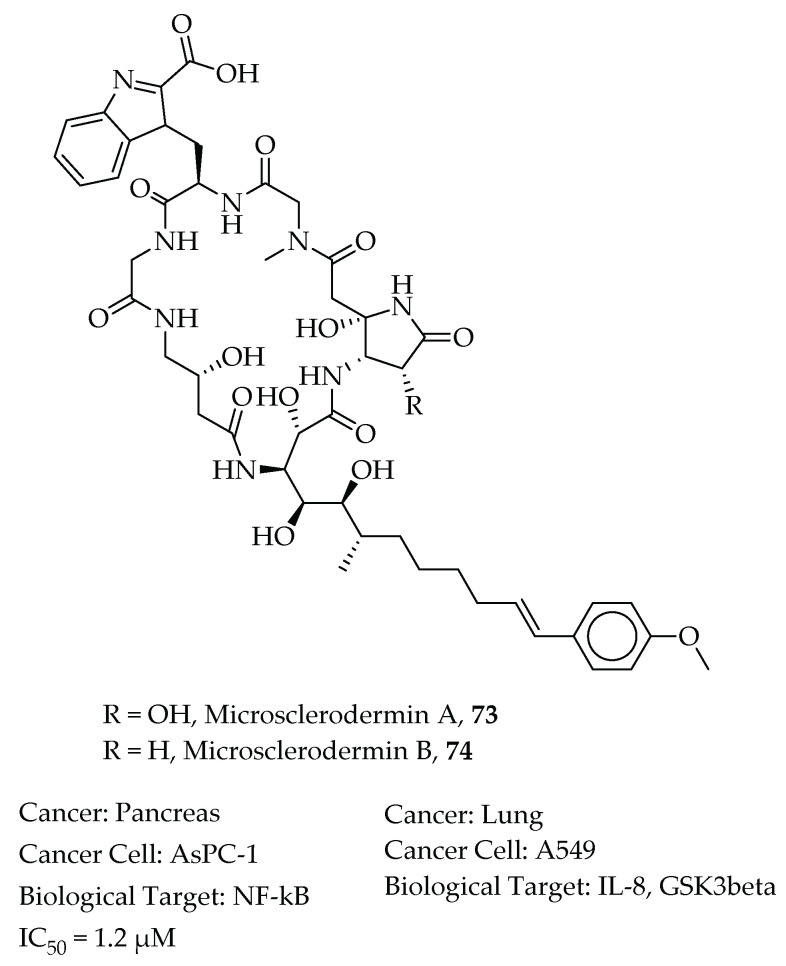
Chemical structures of Microsclerodermin A and B with a summary of their biological activity towards selected cancer targets.

The microsclerodermins are cyclic hexapeptides from a deep-water sponge of the genus *Microscleroderma* [93]. They were first reported by Guzman et al., during their quest for small molecule inhibitors against nuclear factor kappa B (NF-κB) cells [94]. Microsclerodermin A, **73** and B, **74** inhibit NF-κB transcriptional activity leading to reduction of levels of phosphorylated (active) NF-κB in the AsPC-1 cell line, against the AsPC-1, BxPC-3, MIA PaCa-2 and PANC-1 pancreatic cancer cell lines, and induce significant apoptosis in the AsPC-1, BxPC-3 and the PANC-1 cell lines [94]. Subsequent investigation of the mechanism of action of **73** and **74** revealed that they also regulated the expression of proteins in the glycogen synthase kinase 3 pathway (Figure 9) [94].

**Figure 10:**
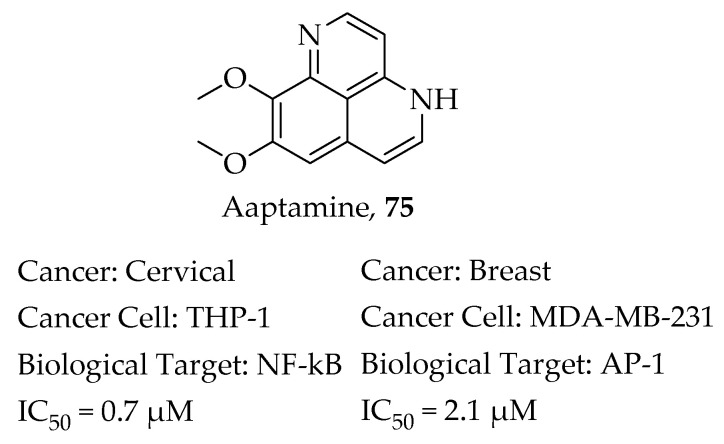
Chemical structure of the benzo[de[1,6]]-naphthyridine alkaloid Aaptamine with a summary of its biological activity towards selected cancer targets.

Aaptamines, **75** are a group of bioactive benzo[de[1,6]]-naphthyridine alkaloids, isolated from marine sponges mostly belonging to the genus *Aaptos* [95]. Aaptamine, **75**, possesses antioxidative, antimicrobial, antifungal, and antiretroviral activity. It is also reported to modulate AP-1, NF-κB, and p53-dependent transcriptional activity in JB6 Cl41 cells (Figure 10) [96].

**Figure 11:**
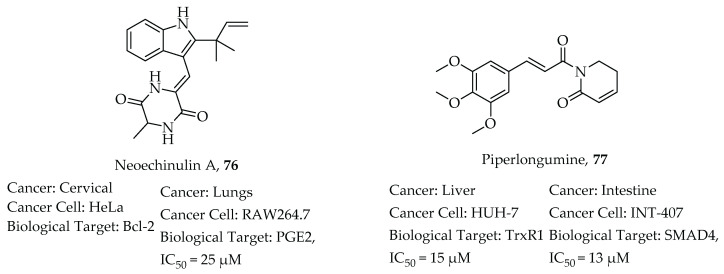
Chemical structures of the amide alkaloids Neoechinulin A and Piperlongumine with a summary of their biological activity towards selected cancer targets.

Neoechinulin A, **76** is from the marine fungal strains *Microsporum* sp and *Aspergillus* sp [97]. It consists of three structural moieties: an indole, a diketopiperazine and an isoprenyl group. Cytotoxicity tests of **76** on human cervical carcinoma HeLa cells showed it to express p53, p21, Bax, Bcl-2, Caspase 9, and Caspase 3 proteins [98]. Western blot analysis also revealed that **76** could induce cell apoptosis through down-regulation of Bcl-2 expression, up-regulation of Bax expression and activation of the caspase-3 pathway [97,98]. Compound **76** also suppressed the production of nitric oxide (NO) and prostaglandin E2 (PGE2) and the expression of inducible nitric oxide synthase (iNOS) and cyclooxygenase-2 (COX-2) [97]. Furthermore, **76** decreased the secretion of pro-inflammatory cytokines, such as tumor necrosis factor-α (TNF-α) and interleukin-1β (IL-1β) [97]. It also blocks the activation of nuclear factor-kappa B (NF-κB) in LPS-stimulated RAW264.7 macrophages by inhibiting the phosphorylation and degradation of inhibitor kappa B (IκB)-α. Moreover, **76** decreased p38 mitogen-activated protein kinase (MAPK) phosphorylation (Figure 11) [97].

Piperlongumine (PL), **77** is an amide alkaloid isolated from long pepper (*Piper longum L.*) [99]. Compound **77** induces the death of numerous cancer cell lines including pancreatic cancer, breast cancer, and leukemia [100,101]. The compound is one of several amide alkaloids from the genus *Piper* that have been found to possess anticancer activities among other biological activities [99,101]. Piperlongumine rapidly induces the death of human pancreatic cancer cells mainly through the induction of ferroptosis [101]. PL directly binds to and inhibits the antioxidant enzyme, glutathione S-transferase Pi 1, resulting in elevated intracellular ROS levels and subsequent apoptotic cell death in cancers. PL has been shown to cause cell cycle arrest [99,101].

Benzophenanthridine alkaloids are commonly found in Papaveraceae and Rutaceae and belong to the benzyl isoquinoline family. They have been extensively investigated for their medicinal properties and bioactive constituents [102]. Though they exhibit a variety of pharmacological functions, only those with multimodality anticancer activities are presented in the current review.

**Figure 12:**
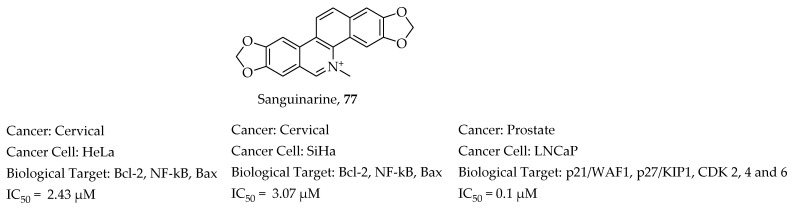
Chemical structure of the quaternary ammonium salt alkaloid Sanguinarine with a summary of its biological activity towards selected cancer targets.

The benzophenanthridine alkaloids are generally isolated from natural sources as their quaternary ammonium salts. Sanguinarine, **78** has been isolated from the roots of *Sanguinaria canadensis*, *Chelidonium majus*, *Argemone mexicana* as well as the roots and leaves of *Zanthoxylum(Fagara xanthoxyloides) zanthoxyloides*, *Z. bungeanum* and *Z. gilletii (Fagara macrophylla).* The compound has diverse medicinal properties including anticancer properties. Although the underlying mechanism of action for Sanguinarine as an anti-tumor agent is yet to be fully elucidated, Xu et al. reported that **78** caused a concentration-dependent inhibition of growth in HeLa and SiHa human cervical cancer with IC_50_ values of 2.43 and 3.07 μM, respectively [103]. Cell cycle analysis revealed apoptosis induction was as a result of downregulation of Bcl-2 and NF-κB and an upregulation of Bax protein expression [103]. Sanguinarine is also reported to cause cell cycle obstruction and induce apoptosis of human prostate carcinoma cells via attenuation of cyclin dependent kinases (Figure 12) [104].

**Figure 13:**
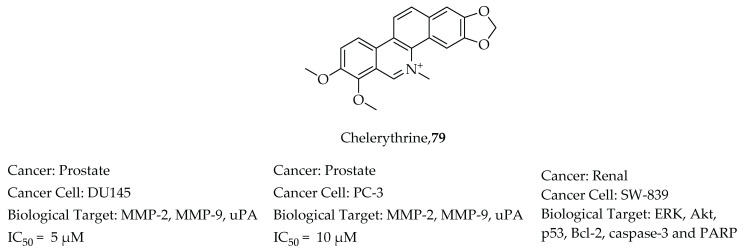
Chemical structure of Chelerythrine with a summary of its biological activity towards selected cancer targets.

Chelerythrine **79**, isolated from *Chelidonium majus*, *Z. xanthoxyloides, Z. clavaherculis,* and *Z. rhoifolium* among others, is a potent, selective, and cell-permeable protein kinase C inhibitor [105]. Chelerythrine exhibits its antitumor properties by inhibiting the proliferation of androgen-independent prostate cancer cells DU145 and PC-3 at concentrations of 5 and 10 μM [106]. Western blot analysis revealed that **79** inhibited metastasis of prostate cancer cells by suppressing MMP-2, MMP-9, and uPA and also boosted the expression of endogenous inhibitors TIMP-1 and TIMP-2 as well as plasminogen activator inhibitors (PAI-1 and PAI-2) [106]. In vitro and in vivo studies have also disclosed the effects of **79** on the growth inhibition and apoptosis induction on HEK-293 and SW-839 renal cancer cells [107]. Chelerythrine, **79**, also significantly inhibits the phosphorylation of extracellular signal-regulated kinase (ERK) and Akt accompanied by upregulation of p53, and downregulation of Bcl-2, caspase-3, and PARP (Figure 13) [107].

**Figure 14:**
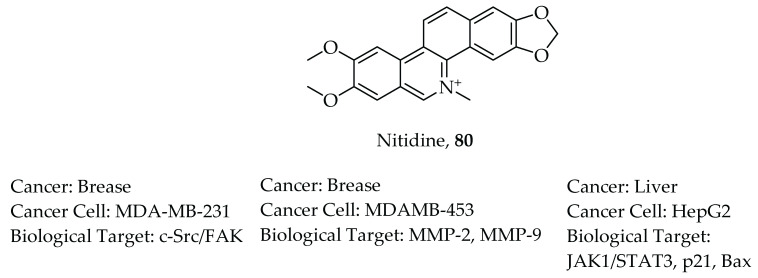
Chemical structure of Nitidine and a summary of its biological activity towards selected cancer targets.

Nitidine **80** is found mostly in the roots of *Zanthoxylum nitidum* (*Fagara nitida*), *Z. gilletii* (*Fagara macrophylla*), *Z. tessmanii*, and *Z. chalybeum* (*Fagara chalybea*). Nitidine was first reported from *Z. nitidum* in 1958 by Arthur et al. [108]. It was later reported from *Z. gilletii* (*Fagara macrophylla*) by Torto and Addae-Mensah in 1970, Wall et al. in 1971, and Fish and Waterman in 1972 [109,110,111,112]. Since then, it has been isolated from other *Zanthoxylum species* as well as *Toddalia asiatica*. It is usually isolated in the form of its chloride salt, and is noted mostly for its antimalarial and cardiovascular activities [113,114,115]. It has also been extensively investigated for its wide variety of bioactivity including its anti-tumor activity against various malignancies. Nitidine is a very strong inhibitor of topoisomerase I enzyme at 0.05–0.3 μM with significant selectivity. However, its effect on topoisomerase II is only moderate [116,117]. DNA topoisomerases are enzymes that regulate the conformational changes in DNA topology by catalyzing the concerted cleavage and polymerization of DNA strands during cellular growth. Recent studies have identified them as putative targets for drug design against various forms of cancers. Nitidine also impedes breast cancer cell migration and invasion via inactivation of c-Src/FAK pathway as well as downregulation of MMP-2 and MMP-9 [118]. An in vivo study revealed the suppression of hepatocellular carcinoma cell proliferation by the inhibition of the JAK1/STAT3 signaling pathways. Associated with this is the increased levels of p21 and Bax as well as decreased levels of cyclinD1, CDK4 and Bcl-2 (Figure 14) [119].

The existence of nitidine and other benzophenanthridine alkaloids as quaternary salts is a major problem in their study for cancer chemotherapy and other diseases, especially in vivo studies, because of their poor absorption and hence bioavailability. In attempts to find possible drug delivery systems to enhance their cancer chemotherapeutic profiles, the pharmacodynamics of nitidine chloride on various types of nanoparticles have been studied and found to reduce toxicity and enhance anticancer effects [120].

**Figure 15:**
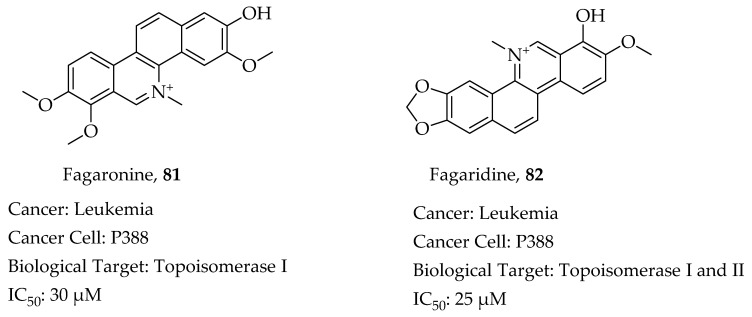
Chemical structures of Fagaronine and Fagaridine with a summary of their biological activity towards selected cancer targets.

Closely related to nitidine but less toxic are fagaronine, **81** and fagaridine, **82**, which are major constituent alkaloids of *Z. xanthoxyloides* (*Fagara xanthoxyloides*). The two compounds have been extensively investigated for their mutagenic activities [121,122]. The anticancer activity of fagaronine was first reported by Messmer and his coworkers, who found it to effect complete cure in mice infected with the P-388 lymphocytic leukemia [123]. It was subsequently found to be very active against chronic myeloid leukemia (cell line K 562) as well as several other cancers of viral origin [124]. Fagaronine exhibits its antileukemic activity by inhibiting both Topoisomerase I and II at concentrations of 30 and 25 μM [125]. Fagaridine, first reported from *Z. xanthoxyloides* by Torto et al., [126] inhibits topoisomerase I, effectively stabilizes topoisomerase I-DNA binary complex, and intercalates into DNA [127]. A synthetic isomer of fagaridine, N-109, is said to be more stable and possess greater activity than all the naturally occurring benzophenanthridine alkaloids (Figure 15) [109].

### 2.2. S-Based Heterocycles 

In addition to the N- and O- based heterocycles, medicinal chemists have shifted their attention to S-based heterocycles because of their impressive biological attributes as anticancer, antidiabetic, antifungal and antihypertensive agents [128]. Currently, some FDA approved drugs have *S*-heterocycles as core moieties for the treatment of various diseases including cancer [128]. Below are some *S*-containing heterocycles with multitarget anticancer properties. 

**Figure 16:**
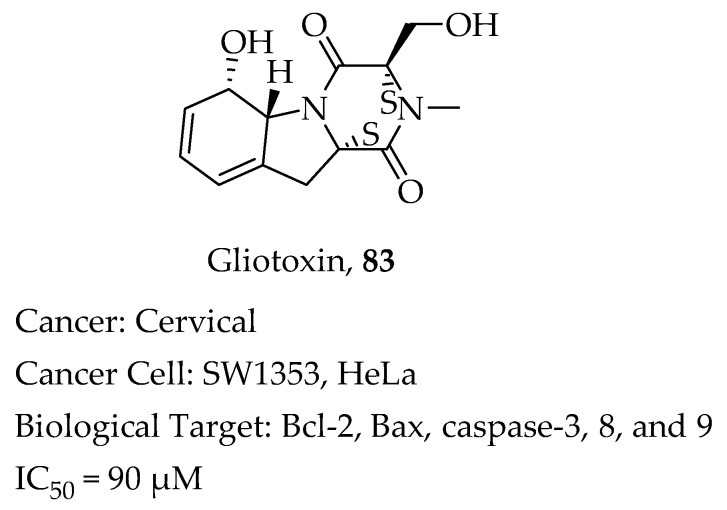
Chemical structure of Gliotoxin and summary of its biological activity towards selected cancer targets.

Gliotoxin, **83** is a member of the epipolythiodioxopiperazine class of mycotoxins produced by *Aspergillus fumigatus, Eurotium chevalieri, Gliocladium fimbriatum,* and most *Trichoderma,* and *Penicillium* species as well as *Neosartorya pseudofischeri* [129]. It belongs to the 2,5-diketopiperazine class of natural products. Compound **83** has immunosuppressive properties such as the blockage of NF-κB activation [130] and modulates the immune response, affects circulating neutrophils, suppresses reactive oxygen species (ROS) production and inhibits phagocytosis of conidia [129]. It inhibits proliferation and induces apoptosis on colorectal and cervical cancer cells via caspase activation followed by up- and downregulations of Bax and Bcl-2, respectively (Figure 16) [131,132].

During investigations aimed at repurposing Nonsteroidal Anti-Inflammatory Drugs (NSAIDs) COX-2 selective inhibitors against MCF-7 (human breast carcinoma), HT-29 (human colorectal adenocarcinoma), and A549 (human lung carcinoma) cancer cell lines, two diphenyl thiazole derivatives **84** and **85** were found to exhibit the highest potency with IC_50_ in the micromolar range [133]. Despite the weak activity of the two compounds against tubulin polymerization assays, they showed remarkable inhibition of EGFR and BRAF with IC_50_ ≤ 40 μM [133].

**Figure 17:**
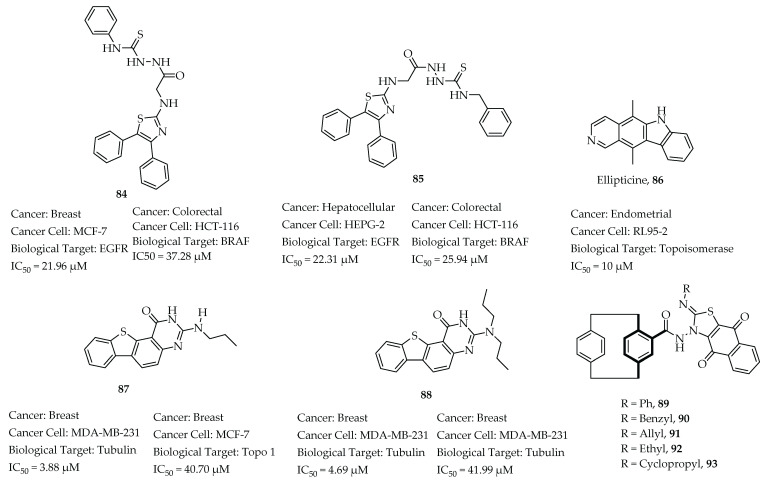
Structures of the diphenyl thiazoles **84** and **85**, ellipticine **86**, benzothienoquinazolinones **87** and **88** with a summary of their biological activity towards selected cancer targets.

In the search for naturally occurring compounds with multitargeting potential against breast cancer cell lines, the alkaloid ellipticine **86** was found to exhibit topoisomerase I and tubulin polymerase inhibition [134]. However, it was found to have numerous deleterious side effects. The continued search for other heterocyclic compounds with activities like that of ellipticine but with reduced deleterious side effects led to the investigation of several heterocyclic compounds containing both nitrogen and sulfur. Six benzothienoquinazolinone derivatives were synthesized via isosteric iteration. Two of the six, compounds **87** and **88**, were the most active compounds inhibiting topoisomerase 1 and tubulin polymerization [135]. The two compounds also exhibited aggressive activity against MDA-MB-231 cell lines with IC_50_ of 3.88 μM and 4.69 μM, respectively and moderate inhibition activity against MCF-7 cells at 40.70 and 41. 99 μM (Figure 17) [135].

The ability of a bioactive compound to quench activities that lead to abnormal cell growth has been applied in the treatment of inflammatory-related diseases. Five highly reactive naphthoquinones (**89**–**93**) investigated by Aly et al. showed promising anti-tumor activity via cell cycle arrest in the pre-G1 and G2/M phases and downregulation of cyclin-dependent kinases (CDK) [136]. Three of them, **91**–**93** were found to be strong inhibitors of cyclic dependent kinases with **91** being the most potent. Compound **91** attenuated eight isoforms of CDK and phosphor-tyr15 and also induced apoptosis and cell cycle arrest by downregulating pre-G1 and G2/M phases, respectively [136]. 

In similar studies involving heterocyclic compounds containing nitrogen and sulphur, ten [1,2,4]-triazine derivatives **94**–**103** (Table 1) were investigated for their anti-cancer properties. The compounds were synthesized from phenylisothiocyanate and ethanol- containing triethylamine via a multi-component reaction. The synthesized compounds were found to be potent at nanomolar and sub-micro concentrations towards six tyrosine kinase receptors (c-Met, c-Kit, Flt-3, VEGFR-2, EGFR, and PDGFR). Compound **95** bound strongly to all the receptors except EGFR [137]. Estimation of the series efficacy against PIM-1 activity showed compounds **95**–**99** and **101**–**103** as the most potent [137].

### 2.3. O-Heterocyclic

**Figure 18:**
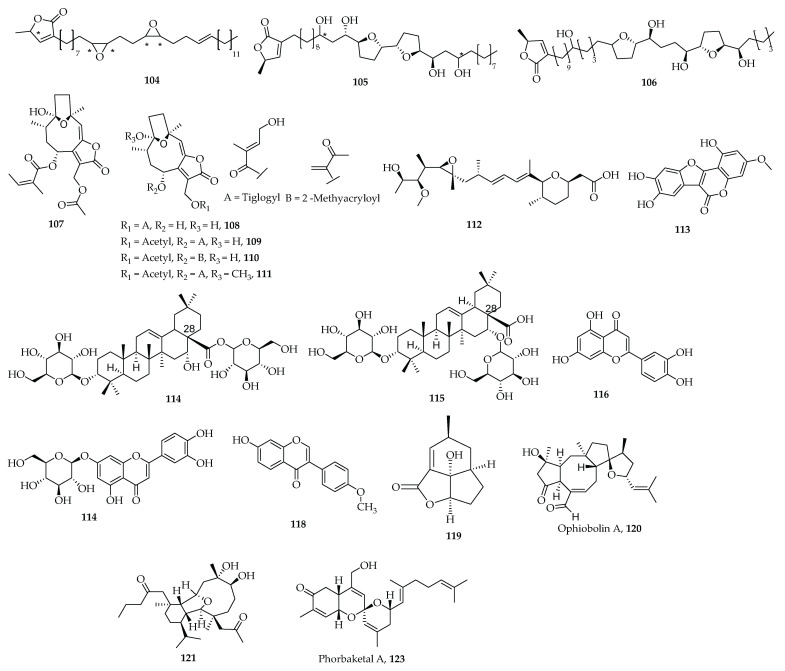
Structures of acetogenins **104**–**106**, sesquiterpene lactones, **107**–**111** and compounds isolated from *Eclipta prostrata* **113**–**117**.

Several synthetic and naturally occurring oxygen heterocycles belonging to various compound classes such as terpenoids, coumarins, flavonoids and acetogenins have been investigated for their anti-cancer activities. Three new naturally occurring acetogenins from the family *Annonaceae,* isolated from the seeds of *Annona squamosa* (compounds **104**–**106**, (Figure 18) have been reported by Pardhasaradhi et al. to possess radical-generating properties against MCF-7 and K-562 [138]. Studies by other researchers revealed that the compounds also induce apoptosis accompanied by organelle deformations like DNA fragmentation and phosphatidyl serine externalization [139].

In the area of cancer chemotherapy, transcriptional factors in modulation target have received little attention. In this regard, Youn et al. investigated the regulation of atypical oncogenes, particularly NF-κB and STAT3 transcriptional factors. They found that the sesquiterpene lactones, **107**–**111** (Figure 18) isolated from *Vernonia cinerea* exhibited promising TNF-α-induced NF-κB and NO activity [140,141]. While compound **109** was the most potent with IC_50_ value of 0.6 µM against TNF-α-induced NF-κB and 2.0 μM against NO targets, the other compounds showed moderate inhibition with IC_50_ values between 10.2 and 13.6 μM [141]. Furthermore, all the sesquiterpene lactone isolates exhibited significant STAT3 aberrations [140]. Similarly, compound **112** was screened against human umbilical vein endothelial cells (HUVECs). It significantly suppressed the vascular endothelial growth factor and downstream hypoxia-inducible factor-1α (HIF-1α), which are used by tumor cells to survive and grow in microenvironments, at a very low concentration of 0.026 µM [142].

Ethnobotanical and phytochemical studies of *Eclipta prostrata* led to the isolation of four compounds **113**–**117** (Figure 18) [143]. Mechanistic studies revealed that **101** activated caspase dependent apoptosis via tight inhibition of 5-LOX at 2.5 µM [143,144]. Compound **114**, which differs from **115** by an additional glycone at the C-28 position, induces apoptosis in a caspase-independent manner via blocking of significant pathways like mammalian target of rapamycin (mTOR) and mitogen-activated protein kinase (MAPK) in human ovarian cancer cells at 94.87 µM [145]. The anticancer property of luteolin, **116**, a tetrahydroflavone, was associated with induction of apoptosis via redox regulation, DNA damage, and protein kinases in inhibiting proliferation of cancer cells [146]. Compound **116** also suppresses metastasis and angiogenesis through downregulation of survival pathways, such as PI3K/Akt, NF-κB, and MAPK [146]. A 7-*O*-glucoside derivative of **116** was found to decrease the proliferation of HepG2 cells along with changes in nuclear structure and DNA fragmentation in a dose dependent manner [147]. The effect of **116** on growth inhibition factors was attributed to the G2/M phase arrest and ROS generation with high phosphorylation of c-Jun *N*-terminal kinases (JNK) [147]. Another multitarget anticancer compound with high selectivity for cell cycle regulatory proteins is the isoflavone **118** [148]. Collectively, compound **118** disrupts activities of cyclin dependent kinases and other cell cycle regulatory proteins resulting in cell cycle arrest with IC_50_ values within the micro-molar range [148]. Similarly, compound **119**, a fungal metabolite with potent antitumor and anti-inflammatory effects with efficacy at 10 µM against prostate cancer cell lines was observed to inhibit both STAT3 and NF-κB transcription and destabilize microtubules and G2/M cell cycle arrest [149,150]. Though the exact mechanism of action of compound **120** is not known, it is reported to have effects on proliferation and migration of human cancer cells. Bury et al. found that 1 µM of compound **120** induced changes in the organization of the actin filaments in the cytoskeleton and disruption of Ca^2+^-activated K^+^ channel activity [151].

The marine environment has been a productive source of potential anticancer agents. One of the most recognized genera as a rich source of eunicellin-based diterpenoids, such as the pachycladins, is *Cladiella* sp. [152]. One such isolate, compound **121** inhibited breast cancer cell growth via down-regulation of the NF-κB and up-regulation of the *Heme oxygenase*-*1* (HO-1) pathways in a dose-responsive manner, with an IC_50_ value of 1.6 μM [152]. Pharmacophoric group analysis indicated that the acetate and butyrate moieties at C-3 and C-11 were responsible for optimal antiproliferative activity [152]. 

Phorbaketal A, a tricyclic sesterterpenoid phorbaketal, **122** isolated from the marine sponge *Phorbas sp* by Yun-Ji Seo was also evaluated for its anticancer potential [153]. It showed significant inhibition of Lipopolysaccharide (LPS)-induced production of nitric oxide (NO) via dampening of inducible nitric oxide synthase (iNOS). It further showed expression at the transcriptional level along with NF-κB deactivation in RAW 264.7 cell [153]. Compound **122** also induced ROS generation through HO-1 expression at 10 µM [153,154].

**Figure 19:**
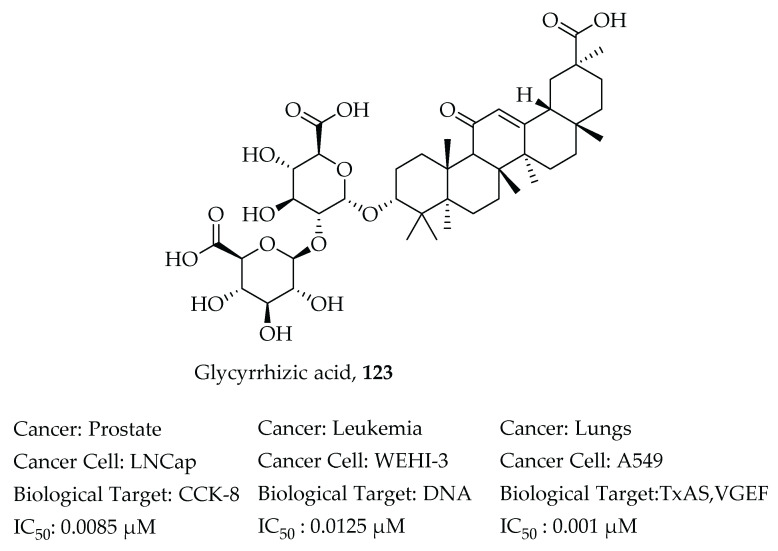
Structure of the triterpenoid glycoside Glycyrrhizic acid with a summary of its biological activity towards selected cancer targets.

*Glycyrrhiza uralensis* Fisch (*Glycyrrhiza glabra* Linn), also known as licorice or Chinese liquorice is one of the most important and oldest phytomedicines in China [155,156,157]. It has been used for ages for the treatment of several diseases, especially those of inflammatory origins. It is also approved as a food additive and various medicinal preparations as a sweetener. Recent attention on the crude drug as well as its chemical components has been on its anticancer properties. It acts by modulating pro-inflammatory mediators and activating the immune system [158,159]. In vitro studies on Glycyrrhizic acid (Glycyrrhizin), **123**, a triterpenoid glycoside from the roots of the plant, showed that the compound downregulates pro-inflammatory modulators NF-κB and thromboxane synthase causing apoptosis on lung adenocarcinoma, hepatoma, leukemia, stomach, and prostate cancer cell lines (Figure 19) [160,161,162]. Its effects on tumor growth and endometrial cancer progression are through suppression of COX-2, TNF-α, IL-1, ornithine decarboxylase (ODC) activity, DNA synthesis, and TxA2 [162,163,164]. The compound also downregulates reactive oxygen species (ROS)-induced damage.

**Figure 20:**
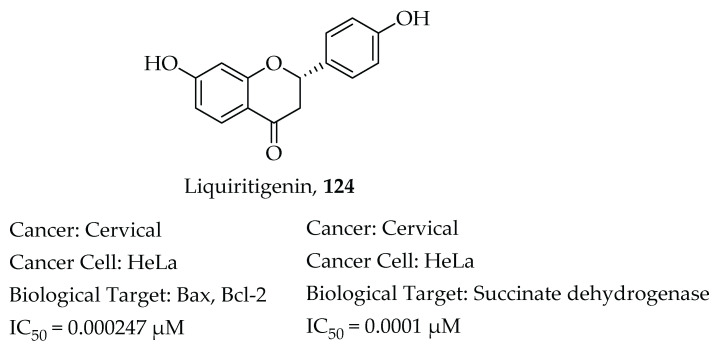
Structure of the dihydroxyflovanone Liquiritigenin with a summary of its biological activity towards selected cancer targets.

A dihydroxyflovanone compound, Liquiritigenin, **124**, isolated from the roots of the same plant [165] is an estrogenic that acts as a selective agonist of the ERβ subtype of the estrogen receptor (ER) (Figure 20) [166]. It is also reported to act as an estrogen receptor alpha (ERα) partial agonist [167]. Liquiritigenin, has been screened against hepatic cancer cell SMMC-7721 and HeLa cells and found to activate apoptosis via MMP-2 activity and dephosphorylation of Akt [156,168].

**Figure 21:**
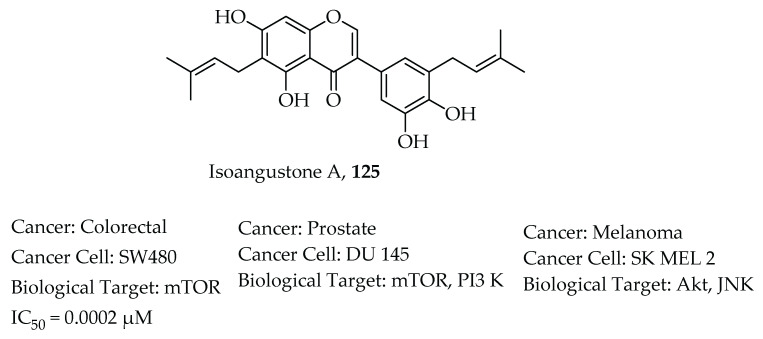
Structure of the 6-prenylated isoflavanone lipid Isoangustone A with a summary of its biological activity towards selected cancer targets.

Isoangustone A, **125** is a 6-prenylated isoflavanone lipid molecule isolated from the root of *Glycyrrhiza* (Figure 21). Compound **125** induces apoptosis in SW480 human colorectal adenocarcinoma cells by disrupting mitochondrial function [169].

The antiproliferation mechanism of **125** has been studied in SW480 human colorectal adenocarcinoma cells [170], prostate cancer cells (PC3, LNCaP, DU 145, and 4 T1) [171,172] and melanoma cells (SK-MEL-2, SK-MEL-5, SK-MEL-28, WM-266-4 cell lines) [171]. Cell cycle arrest and apoptosis via the cessation of cyclin A, cyclin D, cyclin E, CDK2, and CDK4 protein expression and the downregulation of mTOR, PI3 K, Akt, and JNK were the major disrupted pathways identified [171].

**Figure 22:**
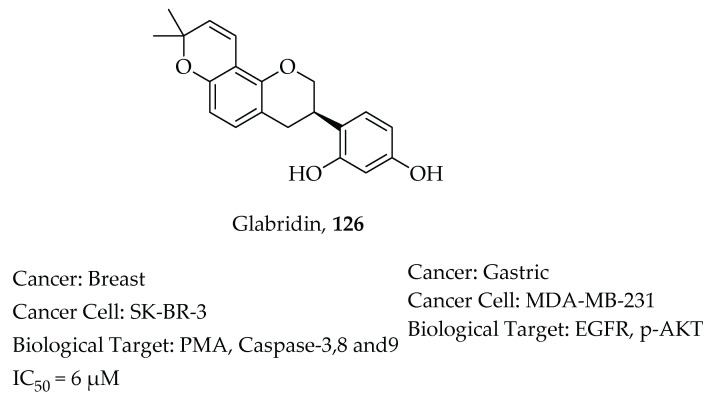
Structure of an isoflavonoid Glabridin with a summary of its biological activity towards selected cancer targets.

Another component of the root of licorice is the isoflavonoid glabridin, **126** from the root of *Glycyrrhiza glabra*. Compound **126** inhibits cyclooxygenase activity and has an anti-inflammatory and an antiplatelet effect (Figure 22) [173].

Studies in modulating metastatic cascade have demonstrated the efficacy of compound **126** via interference of focal adhesion kinase (FAK), proto-oncogene-protein kinase (Src), protein kinase B (Akt), myosin, myosin light chain phosphorylation, and ras homolog family member A (RhoA) with decreased expression and activities of MMP-9, phosphorylation of ERK1/2 and JNK1/2 [174].

**Figure 23:**
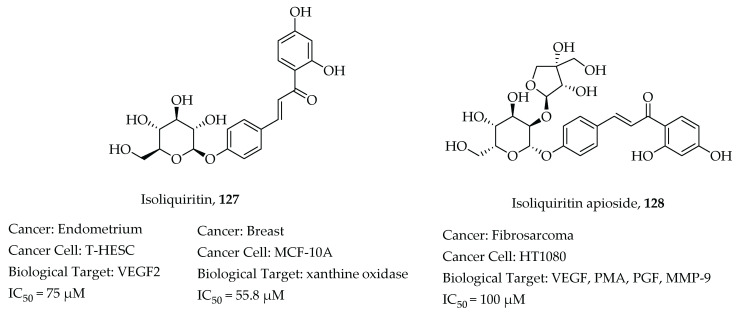
Structures of the flavonoid glycoside Isoliquiritin and its biosynthesized derivative isoliquiritin apioside with a summary of their biological activity towards selected cancer targets.

Isoliquiritin, **127** is a flavonoid glycoside from licorice possessing a broad spectrum of pharmacological activities including antioxidant, anti-inflammatory, and anti-depression activities. Compound **127** and its biosynthesized derivative isoliquiritin apioside, **128**, prevent angiogenesis and tube formation through the inhibition and suppression of pro-angiogenic factors including MMP-9, placental growth factor, and vascular endothelial growth factor under normoxia as well as hypoxia conditions by impairing the hypoxia-inducible factor-1α pathway in HT1080 cells (Figure 23) [175,176].

**Figure 24:**
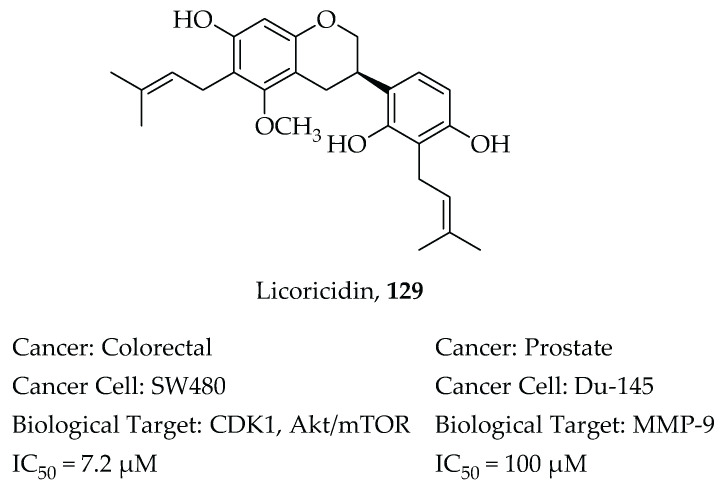
Structure of the prenylated isoflavonoid Licoricidin with a summary of its biological activity towards selected cancer targets.

An isolate from *Glycyrrhiza uralensis,* Licoricidin, **129** is another prenylated isoflavonoid that has been found to inhibit SW480 cells (IC_50_ 7.2 μM) by inducing cycle arrest, apoptosis, and autophagy. It exhibits a range of biological activities including antibacterial, anti-aging, and anticancer activities. It is a potential chemopreventive or chemotherapeutic agent against colorectal cancer (Figure 24) [177]. Compound **129** exhibits its anticarcinogenic effects by inhibiting lung metastasis via suppression of tumor angiogenesis and lymphangiogenesis as well as changing in the local microenvironment of the tumor tissues [178]. It also enhances enhanced gemcitabine-induced cytotoxicity in Osteosarcoma (OS) cells through inactivation of the Akt and NF-κB pathways [179]. The compound also blocks UVA-induced photoaging via ROS scavenging and limits the activity of MMP-1 [180].

Compound **129** has been found to inhibit the migration and adhesion of DU 145 cells in a concentration-dependent manner through the reduction of MMP-9, Urokinase-type Plasminogen Activator (uPA), VEGF, integrin-α2, Intercellular Adhesion Molecule (ICAM), and Vascular Cell Adhesion Molecule (VCAM) secretion [178]. Incubation of glycyrol, **130** against human Jurkat cells, arrests the S phase of the cell cycle by activation of the Fas cell surface death receptor (Fas), caspase-8, and caspase-9 proteins but through the JNK pathway in HCT 116 cells [181].

**Figure 25:**
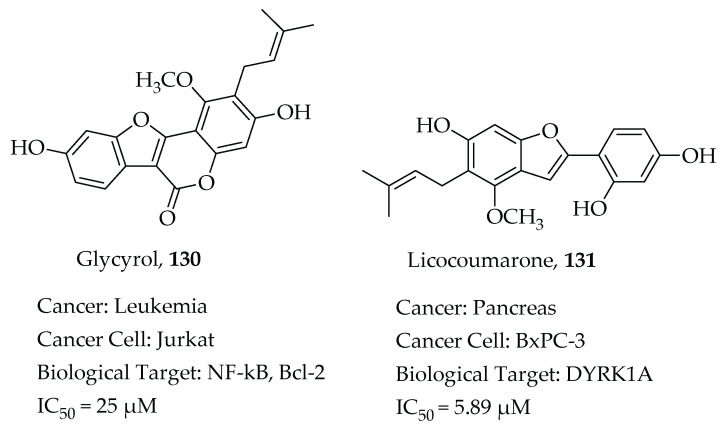
Structures of Glycyrol and Licocoumarone with a summary of their biological activity towards selected cancer targets.

Coumarins are another class of oxygen heterocyclic compounds with diverse bioactivities including anti-cancer activities. Licocoumarone, **131** is one of such compounds isolated from *Glycyrrhiza uralensis*. It acts as an apoptosis-inducing agent [182]. The potency of **131** against cancer cell lines, chromatin condensation and nucleus fragmentation has been investigated and found to induce apoptosis (Figure 25) [183,184].

**Figure 26:**
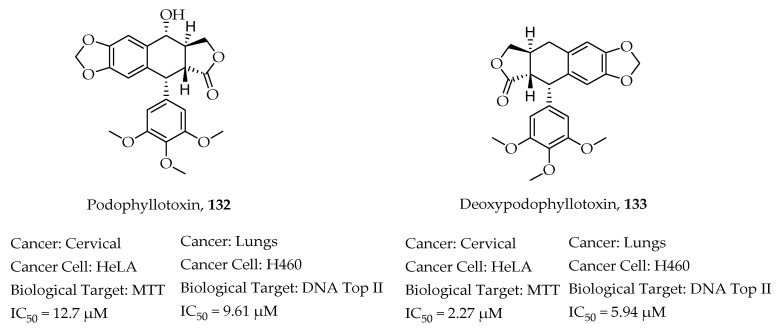
Structure of the cyclolignans Podophyllotoxin and Deoxpodophyllotoxin with a summary of their biological activity towards selected cancer targets.

Recently, the extensive ethnomedicinal use of plants containing lignans has attracted the interest of many natural product and medicinal chemists. Podophyllotoxin (PTOX), **132**, is a lignan produced by various species of *Podophyllum* genus such as *Podophyllum emodi* Wall. (syn. *P. hexandrum*) and *Podophyllum peltatum* L. (Berberidaceae) [185]. Other genera such as *Jeffersonia*, *Diphylleia*, and *Dysosma* (Family Berberidaceae), *Catharanthus* (Apocynaceae), *Polygala* (Polygalaceae), *Anthriscus* (Apiaceae), *Linun* (Linaceae), *Hyptis* (Verbenaceae), *Teucrium*, *Nepeta* and *Thymus* (Labiaceae), *Thuja*, *Juniperus*, *Callitris* and *Thujopsis* (Cupressaceae), *Cassia* (Fabaceae), *Haplophyllum* (Rutaceae), *Commiphora* (Burseraceae), and *Hernandia* (Hernandiaceae) have also been reported to produce podophyllotoxin (PTOX) and its derivatives as well as other lignans [185]. Podophyllotoxin, **132** and deoxy-podophyllotoxin, **133**, both cyclolignans from the *Anthriscus* and *Juniperus* genera have shown excellent therapeutic effect on cancer cells with tubulin, and DNA topoisomerase II as potential targets (Figure 26) [186,187].

Iridoid glycosides are another class of naturally occurring oxygen heterocyclics reported to have a variety of biological activities including antidiabetic, antibiotic, anti-inflammatory, and antioxidant activities. They have been found in several plant species including *Canthium subcordatum, Alchornea cordifolia, Eucommia ulmoides,* and *Gardenia species* [188,189,190,191].

**Figure 27:**
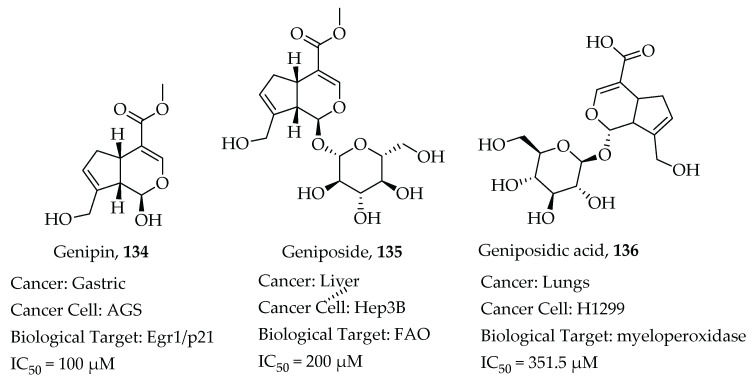
Structure of the iridoid glycosides Genipin, Geniposide and Geniposidic acid with a summary of their biological activity towards selected cancer targets.

Ginipin **134**, Geniposide **135**, and Geniposidic acid **136** are iridoid glycosides found in several plants including *Eucommia ulmoides*, *Gardenia jasminoides*, and *Gardenia fructus* (Figure 27)**.** They possess a range of biological activities including anticancer properties. Their mechanisms of action towards cancer include generation of reactive oxygen species, mitochondria dysfunction and cell-cycle regulation as the leading cause of cell death [192,193]. Particularly, compound **134** causes increased levels of Bax in response to p38 MAPK signaling, leading to the initiation of the mitochondrial death cascade [194] whereas compound **135** inhibits hydroperoxide and myeloperoxidase formation caused by 12-O-tetradecanaoylphorbol-13-acetate (TPA) displacement [195,196].

**Figure 28:**
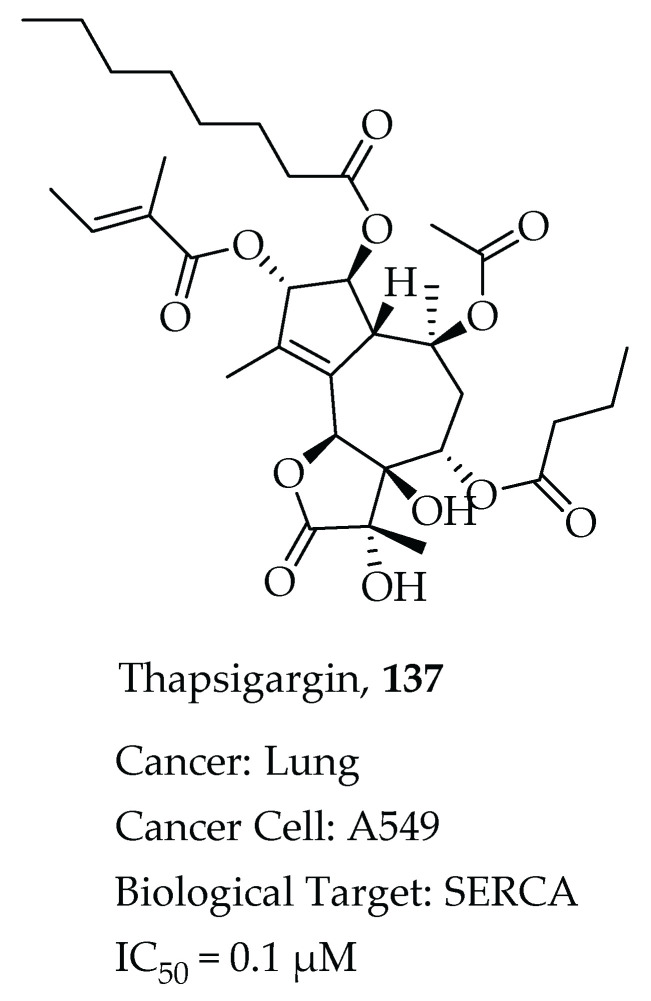
Structure of the sesquiterpene lactone Thapsigargin with a summary of its biological activity towards selected cancer targets.

Thapsigargin, **137** is a sesquiterpene lactone isolated from the umbelliferous plant, *Thapsia gargantea* [197]. It has antitumor activity in the low micromolar range when tested in human cancer cell lines. Cytotoxicity effects are generally attributed to imbalance of calcium homeostasis through interference with sarcoplasmic/endoplasmic reticulum Ca^2+^ ATPase [197]. The compound stimulates MAP kinase signaling via Src and Raf-1. The tumorigenic properties demonstrated by **137** are partially attributed to activation of the Src-MAP kinase pathway [197]. Thapsigargin also induces perturbations in calcium homeostasis and an increase in nitric oxide production eliciting apoptosis and cell death (Figure 28) [198,199,200].

**Figure 29:**
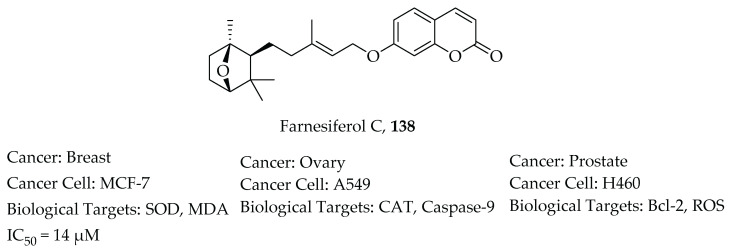
Structure a sesquiterpene coumarin Farnesiferol C with the summary of its biological activity towards selected cancer targets.

Farnesiferol C (FC), **138** is a sesquiterpene coumarin isolated from *Ferula* species (Apiaceae) [201]. It possesses cytotoxic, apoptotic, MDR reversal, antitumor, and antimutagenic properties among other bioactivities (Figure 29)**.** Compound **138** induces cell cycle arrest and apoptosis mediated by oxidative stress in the MCF-7 cell line [202]. It is also reported to deactivate vascular endothelial growth factor (VEGF)-induced cell proliferation, migration, invasion, and tube formation that is the cause of cancer metastasis [202].

**Figure 30:**
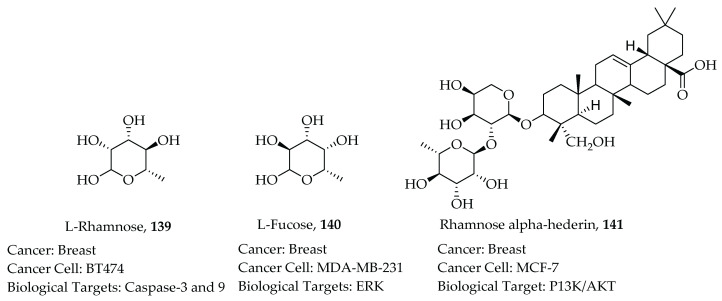
Structures of deoxy sugars L-Rhamnose, L-Fucose, and rhamnose β-hederin with a summary of their biological activity towards selected cancer targets.

Rhamnose is a naturally occurring deoxy sugar that is derived from plants such as Buckthorn, poison sumac, birch trees, and bacteria [203,204,205]. Among the many applications, rhamnose is used in the treatment of skin cancer. L-Rhamnose, **139** and L-fucose, **140** activate apoptosis via the mitochondria pathways particular on the Bcl-2 family proteins [204,205]. In addition, rhamnose α-hederin, **141** is reported to inhibit the 3.5 million PI3K/AKT pathway and activate ERK pathway on breast cancer cells (Figure 30) [206].

**Figure 31:**
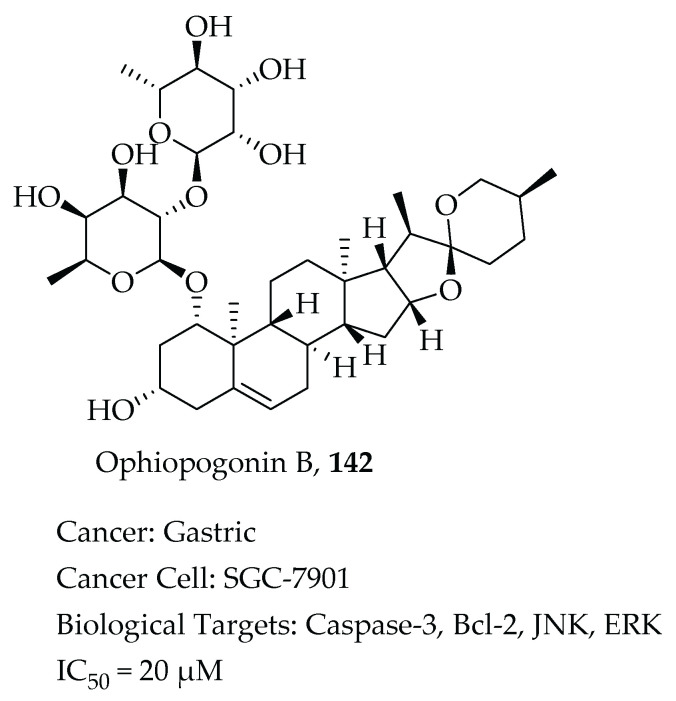
Structure of the saponin Ophiopogonin B with a summary of its biological activity towards selected cancer targets.

Ophiopogonin B, **142** occurs in the herbs *Radix O. japonicus, Ophiopogonis radix* (ophiopogonis root), and *O japonicus* [207]. It is widely used in Chinese traditional medicine. It has a range of anticancer properties against various types of cancer including lung cancer, cervical cancer and gastric cancer [208]. Investigation of changes in intracellular activity of incubated cells with compound **142** using the Western blot method resulted in increased protein expression levels of caspase 3 and B-cell lymphoma 2 (Bcl-2)-associated X protein [208]. Compound **142** also inhibited the proliferation of NPC cells by inducing apoptosis and disturbing the mitochondrial integrity [209]. In addition, compound **142** promotes the expression of mammalian STE20-like kinase 1, large tumor suppressor 1, and phosphorylated-associated protein (YAP), suppresses the expression of YAP and transcriptionally enhances associate domains in NPC cells (Figure 31) [209].

The anti-cancer properties of some oxygen heterocyclics of marine origin and their mechanism of action have also received attention in recent times. The molecular mechanisms by which some of these marine natural products activate apoptosis mainly include dysregulation of the mitochondrial pathway, the activation of caspases, and/or increase of death signals through transmembrane death receptors [210]. Mitogenic roles of VEGF and modulation of the NF-κB pathway have been demonstrated to be the major underlying mechanism of action [211,212,213].

**Figure 32:**
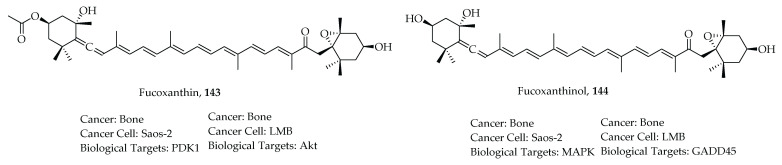
Structures of the non-provitamin A carotenoid Fucoxanthin and its derivative Fucoxanthinol with a summary of their biological activity towards selected cancer targets.

Fucoxanthin, **143** is a naturally occurring brown- or orange-colored pigment that belongs to the class of non-provitamin A carotenoids present in the chloroplasts of brown seaweeds [214]. Anti-proliferative and cancer preventing properties of compound **143** and its derivative fucoxanthinol, **144** are mediated through different signaling pathways, including the caspases, Bcl-2 proteins, MAPK, PI3K/Akt, JAK/STAT, AP-1, GADD45, and several other molecules that are involved in cell cycle arrest, apoptosis, anti-angiogenesis, or inhibition of metastasis (Figure 32) [214]. 

**Figure 33:**
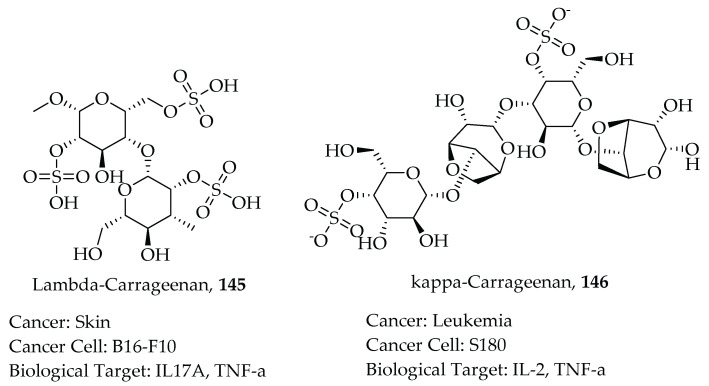
Structures of Sulphated galactopyranosyl Lambda-Carrageenan and Kappa-Carrageenan with a summary of their biological activity towards selected cancer targets.

Carrageenans are linear sulfated polysaccharides extracted from marine red algae, *Kappaphycus striatum* [215]. The coastal region of Senegal and the Gambia, which is the breeding ground for herrings in West Africa, is said to be very rich in red algae, which also serves as food for the young herrings [216,217]. The Senegambian coast is therefore a major source of raw material for the production of carrageenans, which are used as excipients in the food and pharmaceutical industries [217,218,219,220]. However, its use has in recent times elicited considerable controversy in view of its perceived harmful effects. Recent investigations have shown that this class of polysaccharides possesses anticancer properties. The carrageenans consist of alternating 3-linked *β*-d-galactose and 4-linked *a*-d-galactose or 4-linked 3,6-anhydro-d-galactose. The carrageenan oligosaccharides exert their anticancer properties by promoting the immune system [221]. The effects of carrageenan oligosaccharides on transplantable tumors and macrophage phagocytosis, quantitative hemolysis of sheep red blood cells (QHS), lymphocyte proliferation, the activity of natural killer cells (NK), production of interleukin-2 (IL-2) and tumor necrosis factor-a (TNF-a), have been studied [215]. They inhibit the growth of transplantable sarcoma S180 and increase macrophage phagocytosis, spleen lymphocyte proliferation, NK cells activity, serumal-IL-2 and TNF-a level in S180-bearing mice [215,221]. Sulphated galactopyranosyl λ, **145** and κ, **146** carrageenan have been found to inhibit the proliferation of breast, colon, liver, and osteosarcoma cell lines by upregulating proapoptotic factors Caspase-3,9 and 8 with depression of bax/bal-2 ratio (Figure 33) [215,221].

**Figure 34:**
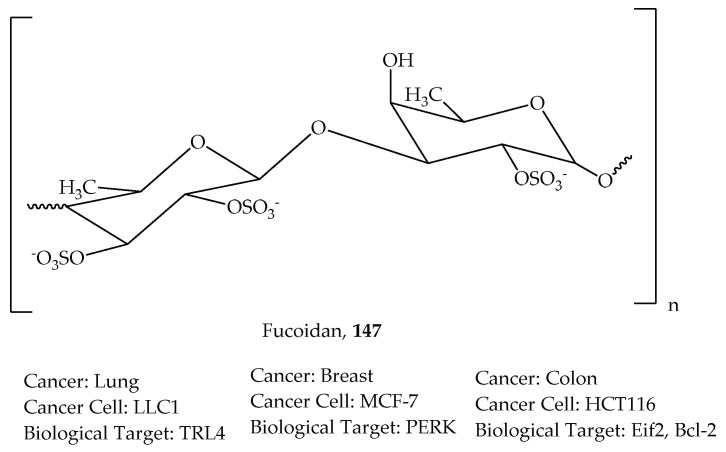
Structure of the polysaccharide Fucoidan with a summary of its biological activity towards selected cancer targets.

Fucoidan, **147** is a polysaccharide largely made up of L-fucose and sulfate groups isolated from brown algae or sea cucumbers, such as *Laminaria digitata*, *Ascophyllum nodosum*, *Macrocystis pyrifera*, *Fucus vesiculosus*, and many others (Figure 34) [222]. Compound **147** exhibits a range of bioactivities including antioxidant, anti-tumor, anti-coagulant, anti-thrombotic, immunoregulatory, anti-viral, and anti-inflammatory effects [222]. The compound inhibits cancer cells through activating apoptosis. It is reported to increase the levels of reactive oxygen species (ROS), and induce an increase in ATF4, CHOP, and ER stress via modulation of Toll-like receptor 4 in lung cancer cells, leading to apoptosis and inhibition of cell proliferation [223]. It is further reported to induce apoptosis in MDA-MB-231 of breast cancer cells as well as activate caspase-8 and -9 in MCF-7 and HeLa cells [224].

**Figure 35:**
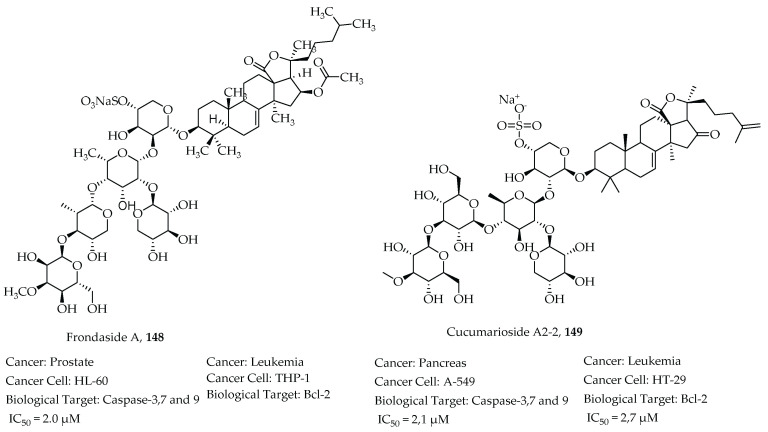
Structures of triterpenoid glycosides Frondaside A and Cucumarioside A2-2 with a summary of their biological activity towards selected cancer targets.

Frondaside A, **148** is a triterpenoid glycoside from the Atlantic Sea Cucumber, *Cucumaria frondose* [225]. Cucumarioside A2-2, **149** is also a triterpene glycoside isolated from the Far-Eastern edible sea cucumber, *Cucumaria japonica* [226]. Both compounds have a broad spectrum of anti-cancer effects, including induction of cellular apoptosis. They inhibit cancer cell growth, migration, invasion, formation of metastases, and angiogenesis [225]. Their cytotoxicity is reported to be through induction of apoptosis by modulation of diverse apoptosis related proteins such as caspase 3, 8, and 9, PARP, and DNA fragmentation [226,227,228]. Both compounds **148** and **149** suppress cell proliferation, increase stress related proteins like the Janus kinase and p38 mitogen-activated protein kinase as well as induce cell cycle arrest via decrease in cell cycle-related proteins, such as cyclin A and cyclin B (Figure 35) [229,230].

**Figure 36:**
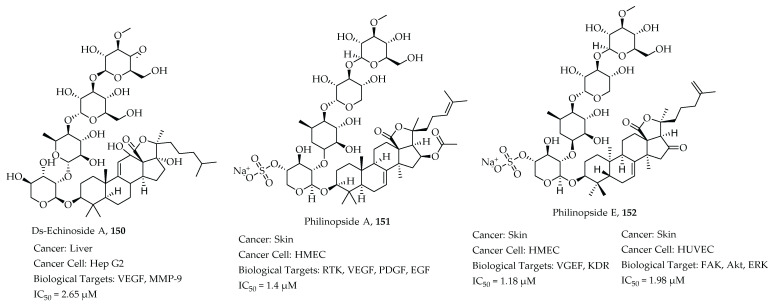
Structures of a non-sulfated triterpene glycoside Ds-Echinoside A and two sulfated saponins Philinopside A and Philinoside E with a summary of their biological activity towards selected cancer targets.

Ds-echinoside A, **150** isolated from *Pearsonothuria graeffei* is a non-sulfated triterpene glycoside derived from the desulfurization reaction of echinoside A [231]. Philinopside A, **151** and Philinopside E, **152** on the other hand are sulfated saponins isolated from the sea cucumber, *Pentacta quadrangulari* (Figure 36) [232,233]. The three compounds all possess a variety of biological activities including antifungal, hemolytic and membranotropic action as well as antiproliferative effects against various human cancer cell lines [234]. They reduce cancer cell adhesion, cell migration, and tube formation [231,235]. Specifically, compounds **151** and **152** reduced the tumor volume by triggering apoptosis of both tumor and tumor-associated endothelial cells via inhibition of angiogenesis-related receptor tyrosine kinases including VEGFR2, FGFR1, and EGFR [232,233]. While compounds **151** and **152** significantly inhibit the proliferation, migration, and tube formation of human microvascular endothelial cells (HMECs) [232,233], **150** on the other hand inhibits the proliferation of human hepatocellular carcinoma cells via MMP9, TIMP-1, and VEGF expression [231].

**Figure 37:**
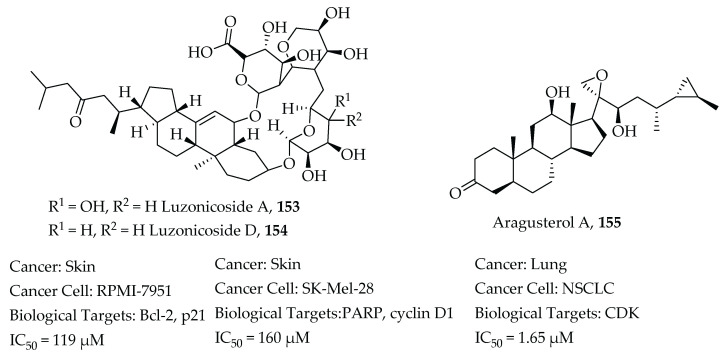
Structures of cyclic steroidal glycosides Luzonicoside A, Luzonicoside D, and a steroidal epoxide Aragusterol A with a summary of their biological activity towards selected cancer targets.

The Luzonicosides (Luzonicoside A), **153** and Luzonicoside D, **154** are cyclic steroidal glycosides from the starfish *Echinaster luzonicus*. They affect cell cycle regulation and apoptosis by inhibiting proliferation, the formation of colonies, and the migration of SK-Mel-28 cells [236]. Aragusterol A, **155** on the other hand is a steroidal epoxide isolated from Okinawan sponge of the genus, *Xestospongia* [237]. It has been established that cell-cycle hindrance by **155** is dependent on the reduced expression of cyclin-dependent kinase (CDK)s and cyclins involved in the G1-S transition such as CDK2, CDK4, cyclin D1, A, and E (Figure 37) [237]. 

**Figure 38:**
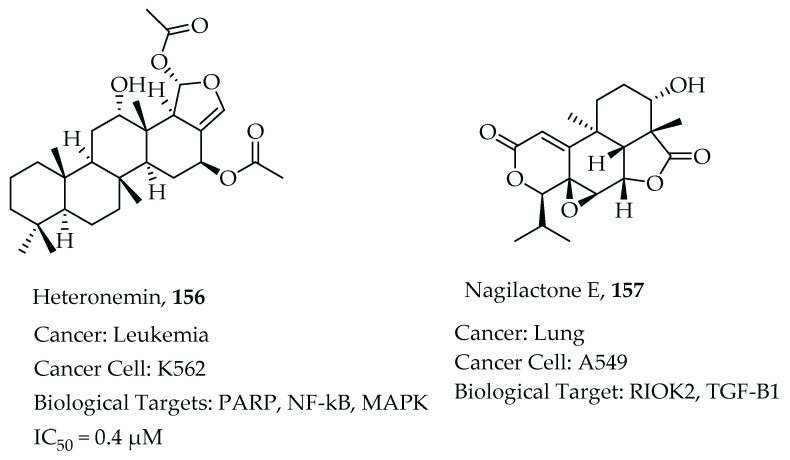
Structures of the sesterterpene Heteronemin and the dinorditerpenoid Nagilactone E with a summary of their biological activity towards selected cancer targets.

Heteronemin, **156** is a sesterterpene isolated from the sponge *Hyrtios sp* [238]. It possesses a pentacyclic scalarane skeleton including a dihydrofuran moiety. Compound **156** induces apoptosis in prostate lymph node carcinoma of the prostate (Lncap) cells via oxidative and ER stress, coupled with the inhibition of topoisomerase II and Hsp90 when evaluated against K652 cells, targeted NF-κB upstream protein mitogen-activated *protein* kinase (MAPK), and engineered tumor necrosis factor (TNF)-α-mediated apoptosis [239]. It also promotes apoptotic cell death by inhibiting the phosphorylation of p38 MAPK (Figure 38) [239].

Nagilactone E, **157**, a dinorditerpenoid isolated from *Podocarpus nagi* [240] upregulates the expression of programmed death-ligand 1 (PD-L1) in lung cancer cells through the activation of *jun* N-terminal kinases (JNK)-c-Jun axis, which has the potential to combine with the PD-1/PD-L1 antibody therapies in lung cancer [240]. It also suppresses transforming growth factor beta 1 (TGF-β1) stimulated cell migration and invasion. It is also a purported protein synthesis inhibitor with strong binding towards serine/threonine-protein kinase (RIOK)2 [241,242]. Furthermore, compound **157** inhibits both cap-dependent and cap-independent translations (Figure 38) [241].

**Figure 39:**
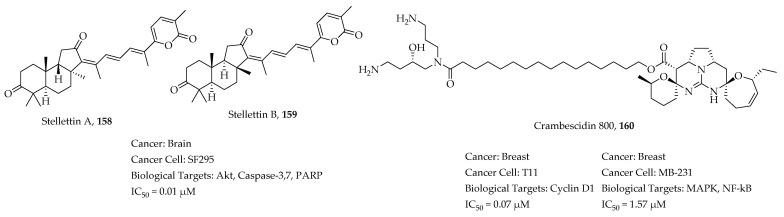
Structures of the isomalabaricane-type terpenoids Stellettin A and B and a pentacyclic guanidine alkaloid Crambescidin 800 with a summary of their biological activity towards selected cancer targets.

Marine sponges are known to be a rich source of bioactive natural products. *Stelletta*, a genus of white marine sponges (Class Demospongiae) is a very rich source of diverse and complex biologically relevant natural products, including alkaloids, terpenoids, peptides, lipids, and steroids. Compounds isolated from this genus include the isomalabaricane-type terpenoids Stelletin A **158**, from the marine sponge *Stelletta tenuis* and Stelletin B, **159** isolated from *Jaspis stellifera*. The compounds trigger cytoprotective activity through the upregulation of proapoptotic Bax-type (Bak and Bax) protein levels, downregulation of Bcl-2 Akt inhibition in A549, K562 and KU812 cells (Figure 39) [243].

**Figure 40:**
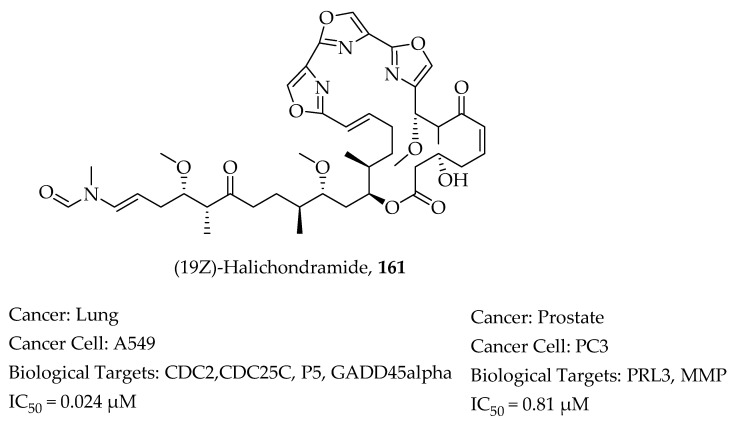
Structure of the trisoxazole macrolide (19Z)-Halichondramide with a summary of its biological activity towards selected cancer targets.

Crambescidin 800, **160**, is a pentacyclic guanidine alkaloid isolated from the marine sponge *Monanchora viridis* [244]. It induces cell cycle arrest and apoptosis in triple-negative breast cancer cells. It also activates p53, which in turn inactivates CDK1, resulting in cell-cycle arrest [244,245,246]. Crambescidin 800 protects HT22 cells against glutamate-induced oxidative toxicity and protects HT22 and neuroblastoma cells from the oxidative stress induced by a hypoxic condition or nitric oxide (NO) (Figure 39) [247]. (19Z)-Halichondramide, **161** is a trisoxazole macrolide from the marine sponge *chondrosia corticate* [245]. It exhibits antiproliferative activity against a variety of cancer cells. For example, it exhibits antimetastatic effect on human prostate cancer cells via modulation of epithelial-to-mesenchymal transition [245]. Cytotoxic activity of compound **161** occurs through Akt/mTOR pathway suppression and G2/M phase is blocked via an increased expression of p53 and GADD45 proteins (Figure 40) [245].

**Figure 41:**
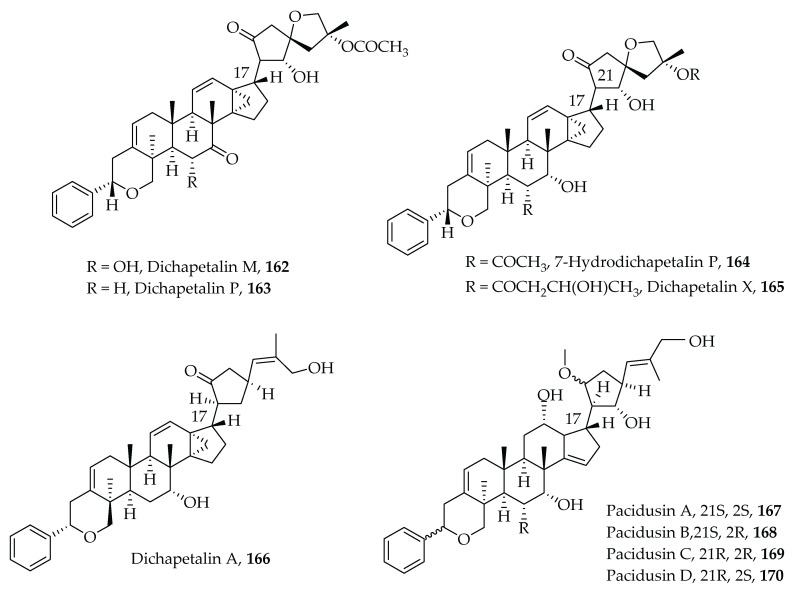
Structures of the triterpenoids Dichapetalins M, P, X and A, 7-Hydroxydichapetalin P, as well as Pacidusins A-D.

The Dichapetalins were first reported as a novel class of triterpenoids from *Dichapetalum madagascariensis* by Addae-Mensah, Achenbach and their co-workers [248,249]. They are mostly found in the family *Dichapetalaceae* (*Chailletiaceae*), but recent reports have confirmed their presence in the *Euphorbiaceae,* particularly, the genus *Phyllanthus* [250,251]. To-date, about twenty-six compounds belonging to this novel class of triterpenes have been reported mainly from various species of *Dichapetalum* and a few from *Phyllanthus*. They are structural derivatives of dammarane characterized by a unique C-6-C-2-unit connection with a variable C-17 side chain mostly containing spirolactone, lactone, lactol, acetal, methyl ester, or furan moieties. The cytotoxic activity of the dichapetalins was first reported by Achenbach et al. who found that dichapetalin A, **165** exhibited very strong in vitro cytotoxicity about seven times greater than that of podophyllotoxin [248]. They also found the compound to inhibit L1210 murine leukemia cells at extremely low doses (EC_90_ < 0.0001 μg/mL but its sensitivity on human KB carcinoma and murine bone marrow stimulated with GM-CSF was at concentrations four-fold higher [248]. Although the cytotoxicity and cancer cell growth inhibition of the dichapetalins are well known, their biological targets of inhibition are yet to be fully elucidated. An investigation by Long et al. assayed the cytotoxic and anti-proliferative properties of ten dichapetalins against human colorectal carcinoma (HCT116) and human melanoma (WM 266-4) cells and found dichapetalins M, **162** and P, **163** as the most potent, displaying activities in the 10^−7^ to 10^−8^ M range [252]. Osei-Safo et al. isolated a novel derivative of dichapetalin P, **164** and two others known dichapetalins X, **165** and A, **166** (Figure 41) from *Dichapetalum pallidum* and investigated their antiproliferative effects against the human T-lymphocytic leukemia (Jurkat), acute promyelocytic leukemia (HL-60) and T-lymphoblast-like leukemia (CEM) cell lines [253]. All the three isolates were significantly bioactive, but dichapetalin X was found to be the most potent against all three cell lines with IC_50_ of 3.14 μM [253]. Similarly, four new dichapetalins (Pacidusin A, B, C, and D) (Figure 41), **167**–**170** were recently isolated from young leaves of *Phyllanthus acidus* and their cytotoxic activities evaluated [254]. While all the isolated dichapetalins showed moderate activity against BEAS-2B and LO2 normal cell lines with IC_50_ less than 22.55 μM, they exhibited strong cytotoxic activities against five human cancer cell lines with IC_50_ ranging between 3.38 and 22.38 μM [254].

**Figure 42:**
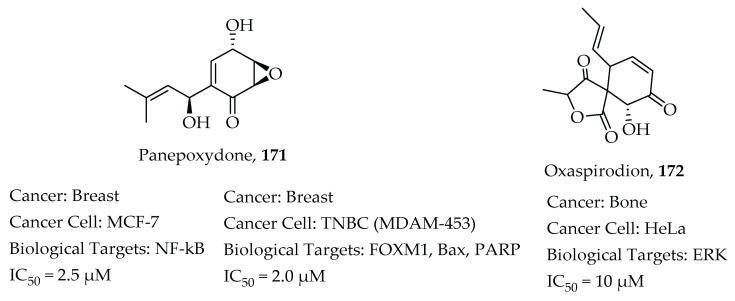
Structures of Panepoxydone and Oxasporidion with a summary of their biological activity towards selected cancer targets.

The nutritional benefits of some species belonging to the Kingdom Fungi such as mushroom, Rhizopus, morels, etc. are enormous and are well documented [255]. Recent studies have also found fungi to possess numerous natural products with diverse medicinal properties for the treatment of infectious and non-infectious diseases [256]. Two potent inhibitors of NF-κB function isolated from the fungus family, panepoxydone, **171** and oxasporidion, **172** induced apoptotic proteins and inhibited TNF-α [257,258]. Compound **171** was isolated from *Lentinus crinitus* (an edible mushroom) while compound **172** was isolated as a mixture of four isomers from fermentations of the ascomycete *Chaetomium subspirale* (Figure 42) [257,258].

**Figure 43:**
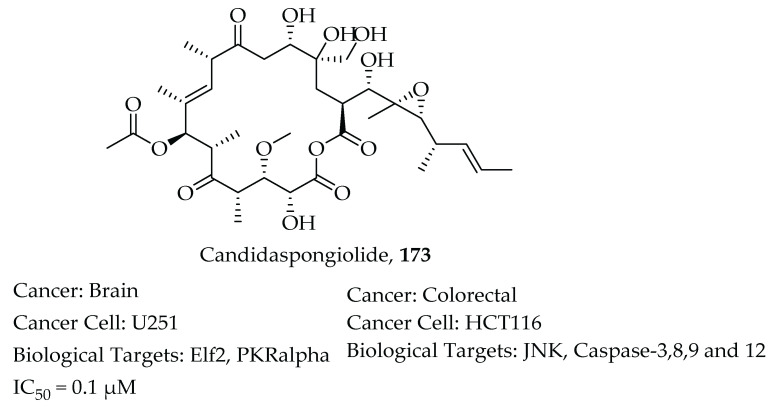
Structure of the polyketide Candidaspongiolide with a summary of its biological activity towards selected cancer targets.

Candidaspongiolide, **173** is a polyketide extracted from *Candidaspongia sp* [259]. It inhibits protein synthesis and induces apoptosis in both U251 and HCT116 cells, the latter in part by a caspase 12—dependent pathway [259]. In addition, compound **173** inhibits proliferation of human melanoma cells in a selective manner compared to breast and lung cancer cell lines (Figure 43) [260].

**Figure 44:**
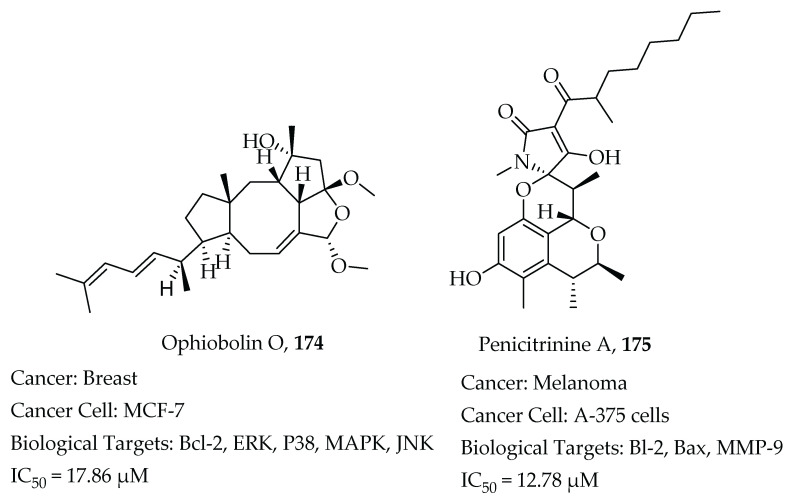
Structures of the sestertepenoid Ophiobolin and the spiro alkaloid Penicitrinine A with a summary of their biological activity towards selected cancer targets.

Ophiobolin O, **174** is a fungal metabolite. It is a sestertepenoid belonging to the ophiobolane group of terpenoids isolated from *Aspergillus ustus* [261]. It is a potent anti-tumor drug for human breast cancer [262]. It induces G1 phase arrest in human breast cancer when tested on the Michigan cancer foundation (MCF)-7 cells and reduces the phosphorylation level of protein kinase B (AKT) and Glycogen synthase kinase-3β (GSK3β). It also induces down-regulation of cyclin D1 [262]. Incubation of **174** with glioblastoma cancer cells resulted in the induction of the death of the glioblastoma cells as a result of G0/G1 cell cycle arrest and Ca^2+^-activated K^+^ channel (BKCa) ion channel activity inhibition (Figure 44) [261,262]. 

Penicitrinine A, **175** is an alkaloid with a unique spiro skeleton, isolated from a marine-derived fungus *Penicillium citrinum.* It showed toxicity against cellosaurus cell line A-375, human cell lines SPC-A1 and HGC-27, and human cancer cell lines with IC_50_ values of 20.1, 28.6 and 29.4 µM, respectively [263]. The anti-metastatic activity is purported to be by decreasing the expression of Bcl-2 and increasing the expression of Bax [263]. Furthermore, compound **175** suppresses metastatic activity of A-375 cells by regulating the expression of matrix metallopeptidase 9 (MMP-9) and its specific tissue inhibitor of metalloproteinase 1 (TIMP-1) (Figure 44) [263].

**Figure 45:**
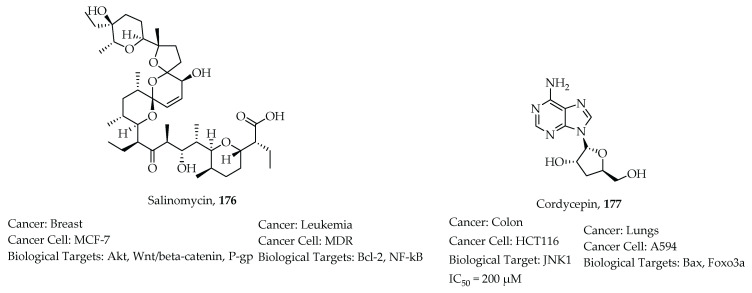
Structures of the monocarboxylic polyether Salinomycin and the nucleoside antimetabolite Cordycepin with a summary of their biological activity towards selected cancer targets.

Salinomycin, **176** is a monocarboxylic polyether antibiotic isolated from *Streptomyces albus* strain (Figure 45) [264,265]. Although the exact mechanism of its anticancer action is unknown, **176** is suspected to decrease the expression of adenosine triphosphate-binding cassette transporter and interfere with the protein kinase B (Akt) signaling pathway [264]. Salinomycin suppresses the phosphorylation of low-density lipoprotein receptor-related protein 6 (LRP6) and the expression of β-catenin and glycogen synthase kinase-*3β* (p-GSK-3β). Additionally, it induces the production of ROS and mitochondrial membrane depolarization resulting in the activation of caspase 3. It also causes the induction of poly(ADP-ribose) polymerase-1 (PARP-1) cleavage, and the elicitation of DNA damage [264]. Consequently, induction of tumor cell death results leading to inhibition of cancer cell growth. The compound also induces autophagic cell death, inhibits the NF-κB pathway and activates p38 mitogen-activated protein kinase (MARK) pathway [266].

Cordycepin, **177**, a nucleoside antimetabolite from *Cordyceps militaris* [267] is an adenosine analogue which is readily phosphorylated to its mono-, di- and tri-phosphate derivatives intracellularly [268]. Compound **177** kills cancer cells resistant to pro-apoptotic stimuli, induces non-apoptotic-related death pathways in cancer cells, and impairs the biology of cancer stem cells (CSCs) [269]. It also causes cell cycle arrest but in the G2/M phase by regulating c-Jun N-terminal kinase activation and TNFα-induced NF-κB activation [267,268].

**Figure 46:**
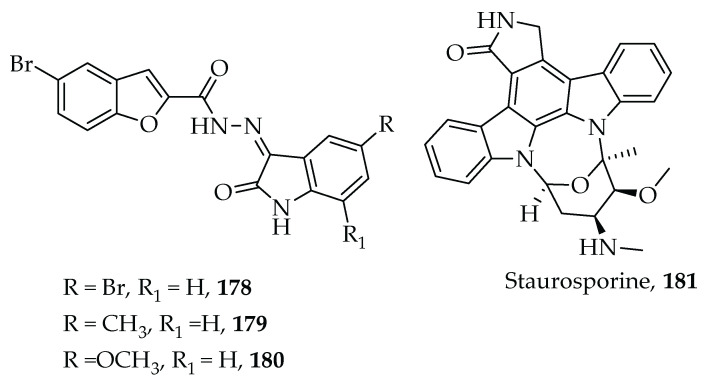
Structures of oxindole-benzofuran hybrids **162**–**164** and the bis-indole Staurosporine with a summary of their biological activity towards selected cancer targets.

Other oxygen heterocyclic compounds which have been screened for multi-target anticancer properties are the oxindole-benzofuran hybrids. Compounds **178**–**180** were found to possess activity against breast cancer when tested against CDK2/GSK-3β. They were found to be more potent in comparison with staurosporine, **181**, a cell permeable alkaloid isolated from *Streptomyces staurosporeus* [270]. The IC_50_ values against MCF-7 cell lines were 3.41 μM (**178**), 3.45 μM (**179**), 2.27 μM (**180**), and 4.81 μM (**179**). When tested against breast cancer cell line T-47D, activities of 3.82, 4.53 and 7.80 μM, for compounds **178**, **179** and **180**, respectively were obtained while that of **181** was 4.34 μM. Compounds **179** and **180** were found to inhibit CDK2/GSK-3β resulting in cell cycle arrest in the G2/M phase (Figure 46) [270].

## 3. Conclusions

Chemotherapy is among the various strategies available for cancer treatment. However, many of the available chemotherapeutic agents suffer serious drawbacks including drug inefficiencies, resistance, and diverse and intertwining pathways of the pathogenesis of cancer diseases. Multi-target drugs have been proposed as the effective means of treating cancers. In this review, different classes of synthetic, semisynthetic, and naturally occurring heterocyclic compounds with multi-targeting properties have been evaluated as potential chemotherapeutic agents for various forms of cancer. The classes of heterocyclics considered in this review are the nitrogen, sulfur, oxygen, combined nitrogen/sulfur, and nitrogen/oxygen heterocyclics. The mode of action of these compounds includes inhibition of cell growth, induction of apoptosis and reduction of cell metastasis via downregulation of topoisomerase I and II, G2/M and G0/G1 cell cycle arrest, HDAC, tubulin polymerization, VEGFR, and EGFR. Some of the heterocyclic compounds also suppress NF-κB, STAT3, P13K/AKT, BRAF, and MDR.

## Figures and Tables

**Table 1:** Inhibitory activity of protein kinase enzymes (IC_50_/nM) by synthesized 1,2,4-triazine derivatives [137].

Compound No.	Cpd	c-Met	c-Kit	Flt-3	VEGFR-2	EGFR	PDGFR	Pim-1
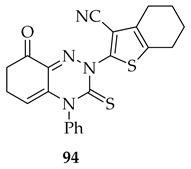	**94**	3.42	0.32	0.24	0.63	0.42	0.57	>10,000
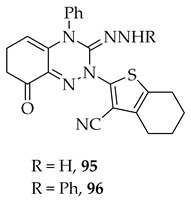	**95** **96**	0.24 0.35	0.23 3.08	0.19 3.16	0.59 5.73	1.03 2.92	0.36 2.73	420 260
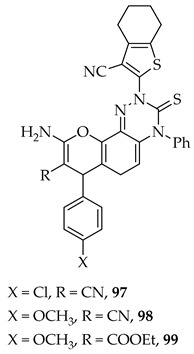	**97** **98** **99**	0.27 0.32 0.48	0.68 0.36 1.17	0.47 0.29 1.34	0.83 0.64 0.93	5.06 0.52 2.53	2.27 0.38 1.03	230 400 >10,000
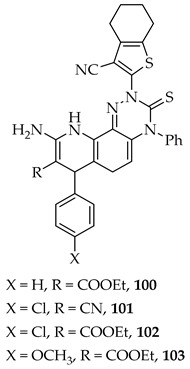	**100** **101** **102** **103**	0.42 0.68 0.49 0.42	0.62 0.52 0.16 0.63	0.49 0.21 0.24 0.51	0.26 0.53 0.37 1.08	0.38 0.80 0.49 0.74	0.41 0.46 0.22 0.80	360 290 >10,000

## Data Availability

Not applicable.
